# Real-world data on oncology in Morocco: a narrative review of four decades of national evidence

**DOI:** 10.1007/s00432-026-06460-6

**Published:** 2026-05-13

**Authors:** Sihame Lkhoyaali, Chaymaa Ghizlane, Badiaa Batlamous, Emmelda N. C. Efountame Nkoghe, Ibrahim El Ghissassi, Hind Mrabti, Hassan Errihani, Mohamed Khalis, Saber Boutayeb

**Affiliations:** 1 Mohammed VI Center for Research and Innovation, Rabat, Morocco; 2 Department of Public Health and Clinical Research, Mohammed VI Center for Research and Innovation, Rabat, Morocco; 3 Mohammed VI University of Sciences and Health (UM6SS), Casablanca, Morocco; 4 Translational Oncology Research Team, Faculty of Medicine and Pharmacy, Mohammed V University, Rabat, Morocco; 5 Medical Oncology Department, National Institute of Oncology, Rabat, Morocco; 6 Faculty of Medicine and Pharmacy of Rabat, Mohammed V University in Rabat, Rabat, Morocco; 7 Mohammed VI International School of Public Health, Mohammed VI University of Sciences and Health, Casablanca, Morocco; 8 Higher Institute of Nursing Professions and Health Techniques, Ministry of Health and Social Protection, Rabat, Morocco

**Keywords:** Real-world data, Oncology, Morocco, Cancer epidemiology, Molecular oncology

## Abstract

**Purpose:**

Real-world data (RWD) has become essential to guide oncology practice and policy. However, in Morocco, the extent and quality of available oncology RWD remain unclear. This study aims to address this gap by examining the current status of RWD in oncology in the Moroccan context.

**Methods:**

We systematically identified and synthesized 308 Moroccan cancer studies (1985–2025) drawn from different electronic databases. Eligible studies included those based on population registries, as well as retrospective and prospective clinical cohorts, molecular and genetic studies, and psycho-oncological or health-system analyses. Data were extracted across five domains: (1) epidemiology, (2) molecular biology, (3) treatment outcomes, (4) survivorship and quality of life, and (5) system-level and public health interventions.

**Results:**

Breast (25%), colorectal (12%), and lung cancers (10%) were the most frequently studied. Most RWD outputs originated from Casablanca (34%), followed by Rabat (27%) and Fez (18%). Clinical and epidemiological studies (56%) were the most predominant study types while molecular papers (21%) highlighted actionable biomarkers: EGFR, KRAS, TP53, BRCA1/2, and VEGF.56

**Conclusion:**

Morocco has transitioned from case-series oncology research to integrated molecular-epidemiologic RWD networks. Strengthening national data interoperability, biobank integration, and linkage between registries and clinical outcomes is now imperative.

**Supplementary Information:**

The online version contains supplementary material available at 10.1007/s00432-026-06460-6.

## Introduction

As defined by the Food and Drug Administration (FDA), RWD in the medical and healthcare field refer to data relating to patient health status and/or the delivery of health care that are routinely collected from a variety of sources (Real-World Evidence | FDA, s. d.). These data come from various sources, including patient registries, electronic health records, administrative claims, pharmacy records, digital health technologies, and social media (Schurman 2025; Real-World Evidence Navigator, s. d.). If subjected to well-chosen statistical analyses, RWD can generate real-world evidence (RWE), which can be used to inform and support healthcare decision making. While clinical trials remain the gold standard for establishing treatment efficacy, the growing accessibility and advancement of artificial intelligence and machine learning methods, along with rising costs and acknowledged limitations of traditional clinical trials, have driven significant interest in the use of RWD to enhance the efficiency of clinical research, accelerate discoveries, and bridge the evidence gap between research and routine practice (Liu and Panagiotakos 2022). In the field of oncology, novel antineoplastic agents are now approved and enter the market without having demonstrated to improve patients’ quality of life or overall survival. Additionally, uncertainties frequently persist with regard to their optimal dosing and how best to combine them with existing interventions, hence the relevance of RWD in capturing such realities (Saesen et al. 2020; Davis et al. 2017).

Real world data generation in Morocco faces structural limitations: fragmented reporting systems, limited electronic health records, and uneven geographic research capacity (morocco-ehealth-country-profile.pdf, s. d.; WHO EMRO—WHO Third global eHealth survey: Atlas of country profiles, s. d.). These structural barriers are particularly consequential in oncology, where the burden of disease and the need for comprehensive data systems are rapidly expanding. In fact, cancer is the second leading cause of death in Morocco, with an estimated 74 000 new cases annually and a projected 70% rise by 2035 (Cancer (IARC), s. d.). The expansion of oncology capacity-anchored by the National Cancer Plan (2009–2025) and the Lalla Salma Foundation’s registry network-has transformed both cancer care and data generation (Plan National de Prévention et de Contrôle du Cancer 2010–2019, s. d.).

This narrative review provides a comprehensive synthesis of Moroccan oncology RWD. It aims to (1) quantify research productivity by region and tumor type, (2) map methodological evolution, (3) highlight key clinical and molecular findings, and (4) identify remaining data and equity gaps.

## Methods

### **Study design and framework**

This work follows a structured narrative review design, guided by the SANRA framework to ensure methodological transparency and scientific reasoning (Baethge et al. 2019). The review adheres to established narrative review standards for scope, selection, and synthesis (Green et al. 2006; Ferrari 2015).

**Table:** 

**Box 1: Definition of concepts**	
**RWD** is defined as data obtained from observational (non-interventional) methodologies normally related to patient health status and/or the delivery of health care. Can be routinely collected from various sources such as registries, EHRs, claims, patient-generated data, biomarker data and wearable devices.	
**RWE** is considered evidence (by scientific methods) generated from RWD.	

### Search strategy and information sources

The review **searched** all Moroccan oncology studies **covering all available study periods.** Databases included PubMed, institutional repositories of the National Institute of Oncology (INO Rabat), CHU Casablanca, CHU Fez, CHU Marrakech. Manual searches were conducted in French and English using keywords: *case-control studies*,* cohort studies*,* longitudinal studies*,* retrospective studies*,* observational studies*,* follow-up studies*,* real-world data*,* real-world evidence*,* oncology*,* cancer*,* carcinoma*,* malignancy*,* Morocco, Maroc.* Complete search strategy and references will be found in supplementary data.

Studies were eligible for inclusion if they were conducted in Morocco and involved a study population composed of Moroccan patients. Only observational or registry-based designs reflecting real-world clinical practice were considered. Eligible studies were required to clearly specify their data source, such as institutional hospital registries, regional or national cancer registries, molecular pathology laboratories, or national health surveys. Each study had to address at least one of the predefined thematic domains: epidemiology and cancer registry reporting, molecular or translational oncology, clinical outcomes and survivorship, quality of life and psycho-oncology, or health-system organization and policy evaluation. We excluded animal or pre-clinical research, narrative editorials, non-oncologic studies, and review articles lacking original Moroccan patient-level data.

### Study selection

Identified studies were imported into Rayyan (Rayyan: AI-Powered Systematic Review Management Platform, s. d.), duplicates were removed, and titles and abstracts were screened independently by two reviewers (CG, BB) using blind mode to minimize bias. Studies meeting the inclusion criteria were retained. Conflicts were resolved through discussion after the blind mode was deactivated. The full texts were subsequently screened via Zotero and the corresponding articles were subsequently retrieved by CG, BB, and EE. (*Zotero | Your personal research assistant*, s. d.)

### Data extraction and synthesis

A standardized extraction sheet captured: publication year, location, cancer site, study design, sample size, primary endpoints, and key molecular or clinical results. Each study was independently categorized into one or more RWD domains. Descriptive statistics summarized frequencies and proportions; temporal and geographic patterns were visualized through heatmaps and trend graphs.

### Quality appraisal

Quality of RWD reporting was graded as high, moderate, or low based on: explicitness of data source, availability of outcome measures. Studies lacking methodological transparency were flagged but included to preserve longitudinal coverage.

### Synthesis strategy

Thematic synthesis integrated quantitative (registry and molecular frequencies) and qualitative (health-system, psycho-oncology) findings. Inter-regional comparisons were made to assess geographic equity of research production and patient representation. Data visualization was conducted using aggregated counts to generate publication trends, cancer-site distribution, and regional productivity maps using R version 4.4.2 (*R 4.4.2*, s. d.).

### Ethical considerations

All included studies had previously obtained local institutional approval. No new patient-level data was collected.

## Results

### National output and temporal evolution

**Fig. 1:**
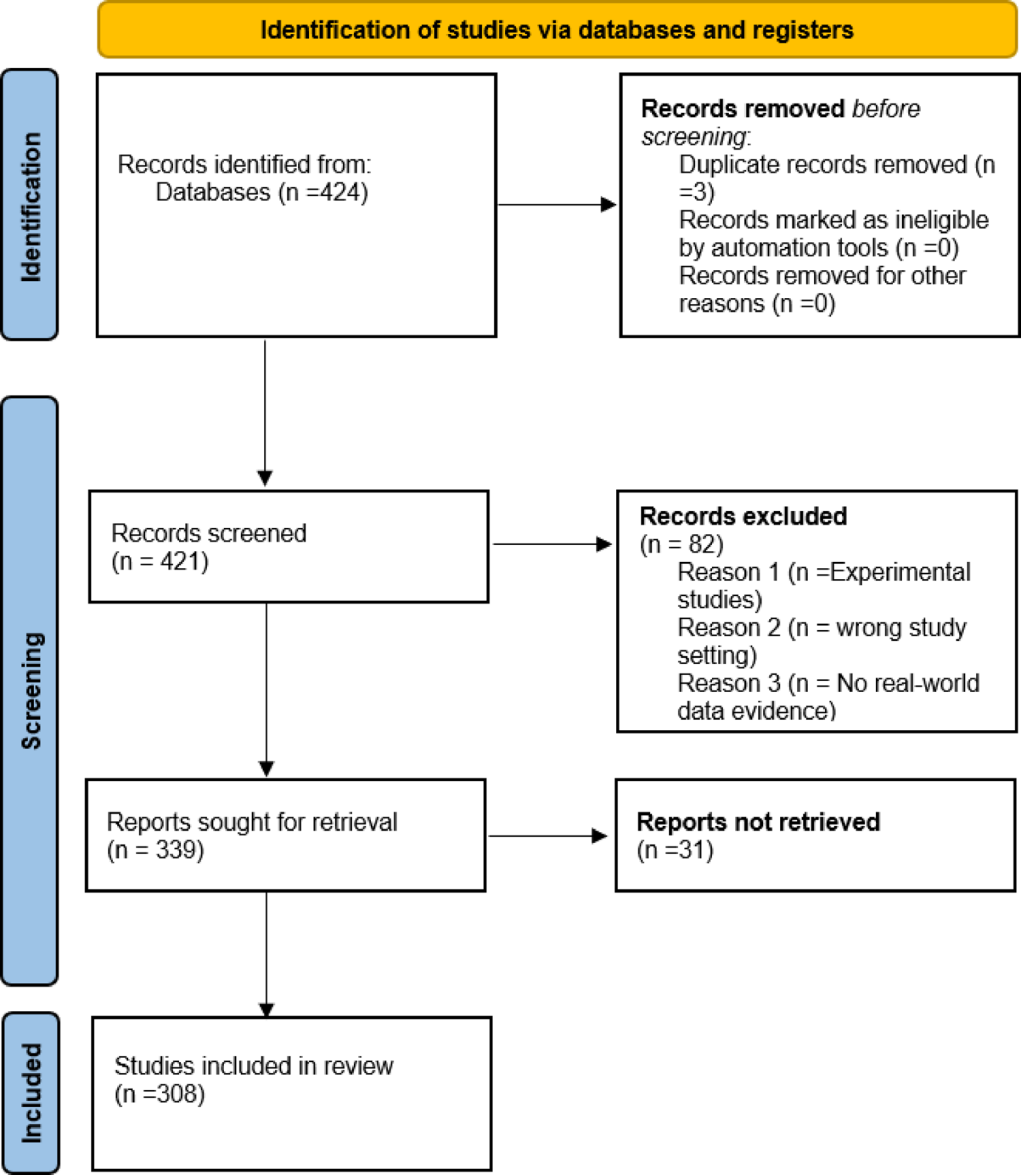
PRISMA flow diagram for included studies

**Fig. 2:**
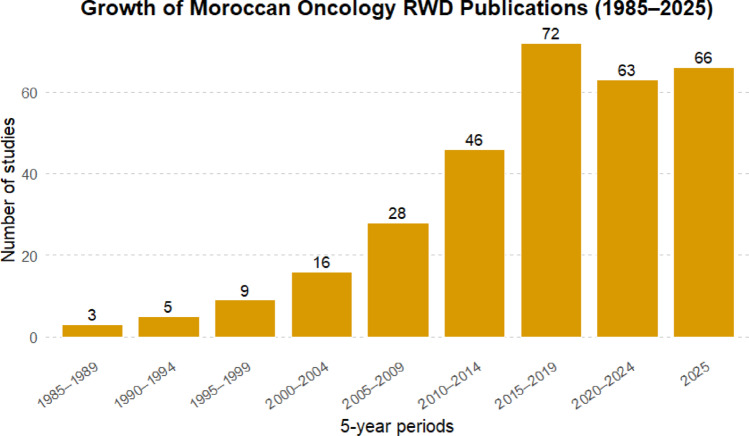
Growth of Moroccan Oncology RWD Publications (1985–2025)

Morocco produced 308 real-world oncology studies between January 1985 and October 2025, with an inflection point after the National Cancer Plan launch (2009–2025) (PNPCC_-_Axes_strategiques_et_mesures_2010–2019.pdf, s. d.; Bouchbika et al. 2013a). Output accelerated through 2015–2024, reflecting multi-institutional cohorts, molecular diagnostics scale-up, and system-level evaluations (Evolution of patterns of care for women with cervical cancer in Morocco over a decade|BMC Cancer|Full Text, s. d.; Tazi et al. 2013a; Elidrissi Errahhali et al. 2017; Morjani et al. 2024; El Koubaiti et al. 2022; El Zaitouni et al. 2024; Mrabti et al. 2021; Radiotherapy of nasopharyngeal cancer using Rapidarc: dosimetric study of military teaching hospital Mohamed V, Morocco|BMC Research Notes|Full Text, s. d.) (Figs. 1, 2).

Publication output in Moroccan oncology shows steady but low activity before 2009, dominated by descriptive hospital series and early registry pilots. A marked inflection occurs after 2010, coinciding with the implementation of the National Cancer Plan (2009–2025) and the establishment of the Casablanca and Rabat registries, leading to the emergence of structured cohort designs and the first molecular epidemiology studies.

The 2015–2019 period represents the peak of productivity ( 72 publications), driven by registry consolidation, routine integration of molecular testing, and expansion of multicenter collaborations.

Despite the COVID-19 pandemic, output remained robust through 2020–2024 ( 63 publications), supported by digital workflows, local biobanks, and adaptive follow-up models.

Early data for 2025 already include more than 60 studies, suggesting a continuing high-productivity phase characterized by translational research, immuno-oncology, and health-system evaluation papers.

This sustained trajectory confirms Morocco’s transition from isolated descriptive reports to an integrated, data-driven oncology research ecosystem (Lamtai et al. 2022; Rais et al. 2020).

### Regional distribution of research productivity

Five poles dominate Moroccan oncology RWD: Casablanca- Mohammedia (34%) reflecting the combined influence of the Casablanca Cancer Registry and CHU Ibn Rochd’s long-standing clinical and molecular activity. Rabat-Salé (27%) driven by the National Institute of Oncology and affiliated military and research units. Fez-Meknès (18%) confirming its rise as a stable third center of molecular and pathological oncology research. Marrakech-Safi (9%), and Oujda-Oriental (6%) have consolidated their regional outputs through registry development and multicenter collaborations; northern coastal and southern regions contribute the remainder (6%) where publication activity remains modest but shows emerging capacity in epidemiologic and radiotherapy reporting (PNPCC_-_Axes_strategiques_et_mesures_2010–2019.pdf, s. d.; Tazi et al. 2013a; El Koubaiti et al. 2022; Mrabti et al. 2021; Ouzzif et al. 2023).

A Casablanca-Rabat corridor accounts for > 60% of national outputs: where tertiary referral centers, molecular platforms, and large registries are concentrated(Bouchbika et al. 2013a),(Mezouri et al. 2014),(M et al., 2015). Fez has emerged as a sustained third hub, with Marrakech and Oujda now contributing consistent hospital-based cohorts. Under-represented regions (northwest and far south) remain priorities for registry expansion and data-linkage pilots(El Koubaiti et al. 2022),(Mrabti et al. 2021),(Derkaoui et al. 2016),(Elidrissi Errahhali et al. 2016). See supplementary data (Fig. S1).

### Cancer-type distribution across Moroccan RWD

**Fig. 3:**
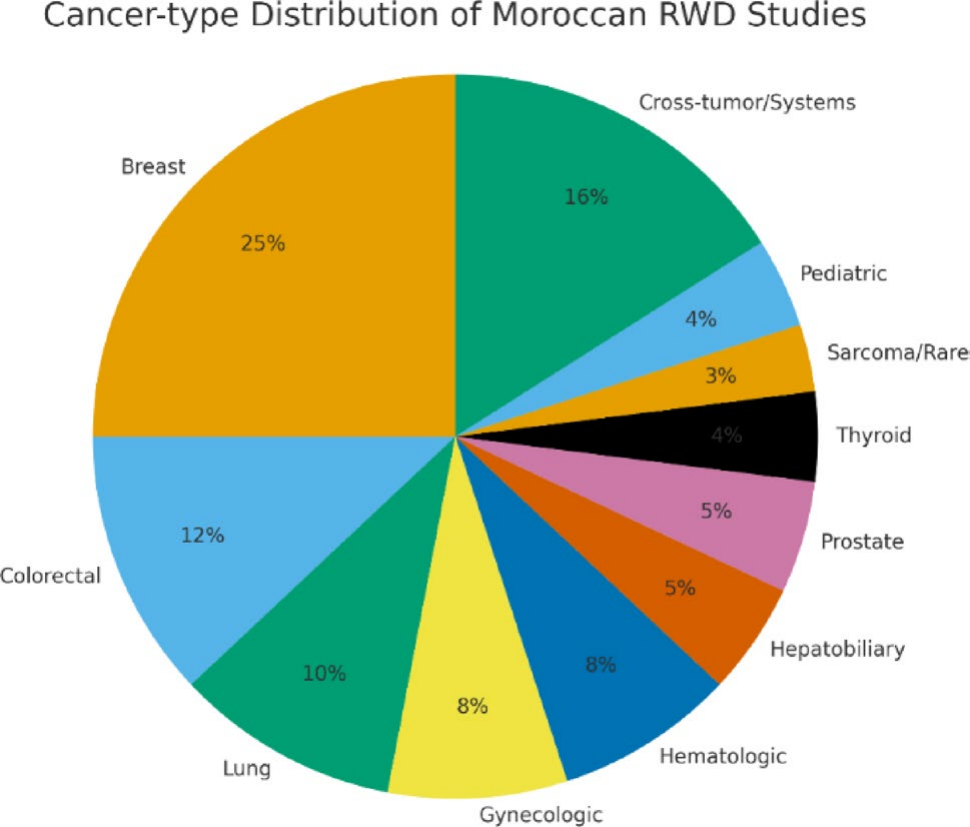
Cancer-type distribution of Moroccan RWD studies

The distribution of topics in the 308 studies is anchored by breast (25%), colorectal (12%), and lung (10%), followed by gynecologic (8%), hematologic (8%), hepatobiliary (5%), and several smaller domains (prostate, thyroid, pediatric, sarcoma/rare) (Tazi et al. 2013a; El Koubaiti et al. 2022; Mrabti et al. 2021; Derkaoui et al. 2016; Elidrissi Errahhali et al. 2016; Psychometric validation of the Moroccan version of the EORTC QLQ-C30 in colorectal Cancer patients: cross-sectional study and systematic literature review | BMC Cancer | Full Text, s. d.; Sbitti et al. 2011; Chibani et al. 2022; Rais et al., s. d.; Khadrouf et al. 2025; Morjani et al. 2024; Qarmiche et al. 2023; El Kinany et al. 2025; Hatime et al. 2022). Cross-tumor and system studies (drug shortages, costs, LTFU, QoL, PROMs) represent 16% (Lamtai et al. 2022; Cheikh et al. 2016a; Errihani et al. 2010) (Fig. 3).

Disease-site attention mirrors service volumes and NGO-supported screening in women’s cancers, with a second wave in CRC and NSCLC tracking the uptake of RAS/EGFR testing and biologics. Pediatric, prostate, thyroid, and sarcoma/rare entities remain comparatively under-represented-prime targets for registry linkage and multicenter consortia.

**Fig. 4:**
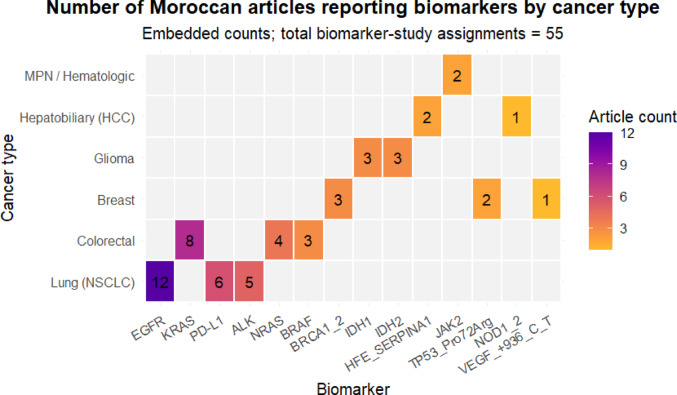
Number of Moroccan articles reporting biomarkers by cancer type Abbreviations : EGFR : Epidermal Growth Factor Receptor. NSCLC : Non-small-cell lung cancer. KRAS : v-Ki-ras2 Kirsten rat sarcoma viral oncogene homolog. CRC : colorectal cancer. BRAF : v-Raf murine sarcoma viral oncogene homolog B1. EORTC QLQ C30: European Organisation for Research and Treatment of Cancer Quality of Life Questionnaire Core 30. HLA : Human Leukocyte Antigen. BRCA1: Breast cancer gene 1. ALK : Anaplastic lymphoma kinase. PD-L1: Programmed Death-Ligand 1. NRAS : Neuroblastoma RAS viral oncogene homolog. HFE : Homeostatic Iron Regulator. SERPINA1: Serpin Family A Member 1. NOD1: Nucleotide-binding Oligomerization Domain Containing 1. NOD2: Nucleotide-binding Oligomerization Domain Containing 2. CTNNB1 : Catenin Beta 1. TP53 : Tumor Protein p53. HCC: Hepatocellular carcinoma. HPV: Human Papillomavirus. CML : Chronic Myeloid Leukemia. MRD : Minimal Residual Disease

- Breast cancer (25%): Moroccan series show younger median age, high triple-negative fraction, and validated EORTC QLQ-C30 tools (Psychometric validation of the Moroccan version of the EORTC QLQ-C30 in colorectal Cancer patients: cross-sectional study and systematic literature review | BMC Cancer | Full Text, s. d.; Derkaoui et al. 2016; BM et al. 2018). Trastuzumab cardiotoxicity and sexual function analyses demonstrate robust survivorship RWD and male breast series provide rare RWD benchmarks (Aitelhaj et al. 2013; Laabadi et al. 2013).
- Colorectal cancer (12%): Molecular cohorts document KRAS: 40–45%, NRAS = 4%, BRAF = 2–5%; bevacizumab outcomes; bevacizumab outcomes and diet/inactivity associations are consistent with regional case-control data (E l Zaitouni et al. 2024; Qarmiche et al. 2023; Sekal et al. 2015; El Agy et al. 2024; Majbar et al. 2022; Imad et al. 2019).
- Lung cancer (10%): EGFR-activating mutations (22%) with female/non-smoker enrichment; ALK and PD-L1 testing integrated since 2018 (chemo-doublet outcomes (median OS approximately 15 months) and TKI-sensitive subgroups) (Clinicopathological and prognostic implications of EGFR mutations subtypes in Moroccan non-small cell lung cancer patients: A first report | PLOS One, s. d.; Boustany et al. 2023; Tafenzi et al. 2024; El Yacoubi et al. 2020).
- Gynecologic cancers (8%): cervical cohorts show HPV-16/18 dominance; delay in diagnosis pre-2010 with measurable post-plan improvement; strong psycho-social and religiosity components documented in recent cohorts (Mezzoug et al. 2025; Berraho et al. 2012; Berraho et al. 2017; Kriya et al. 2025).
- Hematologic malignancies (8%): CML MRD monitoring feasibility(Moumen et al. 2015); AHSCT without cryopreservation validated as cost-saving (Moumen et al. 2015); thalassemia alloimmunization risks characterized (El Kababi et al. 2019).
- Hepatobiliary cancers (5%): Polymorphisms in HFE, SERPINA1, NOD1/2, CTNNB1, TP53 linked to HCC susceptibility (Ezzikouri et al. 2008; Ezzikouri et al. 2010; Zerrad et al. 2024) (Fig. 4).

### Methodological and thematic composition

The corpus is predominantly composed of clinical and epidemiologic investigations (56%), followed by molecular and translational studies (21%), health-system and policy analyses (16%), and quality-of-life or psycho-oncology research (7%).

**Fig. 5:**
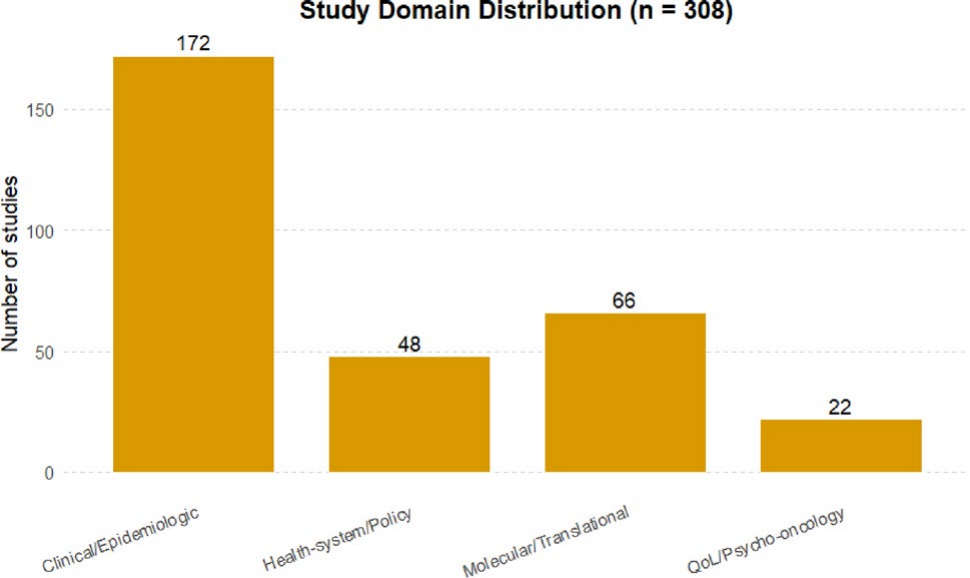
Study domain distribution (*n* = 308)

This distribution reflects the consolidation of outcome-oriented research as the backbone of Moroccan real-world oncology data, while the growing proportion of molecular and system-level studies signals a progressive shift toward integrated, multidisciplinary approaches(Evolution of patterns of care for women with cervical cancer in Morocco over a decade | BMC Cancer | Full Text, s. d.; Tazi et al. 2013a; Elidrissi Errahhali et al. 2017; El Zaitouni et al. 2024; Lamtai et al. 2022; Derkaoui et al. 2016; Cheikh et al. 2016b) (Fig. 5).

The post-2015 era shows a rise in molecular papers (EGFR, KRAS/NRAS/BRAF, TP53, BRCA1/2, VEGF, HLA, inflammatory cytokines), paralleled by system-level analyses (lost-to-follow-up, cost of care, drug-shortage pharmacovigilance, SBRT adoption, PROMs validation), which are essential to policy translation (Clinicopathological and prognostic implications of EGFR mutations subtypes in Moroccan non-small cell lung cancer patients: A first report | PLOS One, s. d.; El Zaitouni et al. 2024; Lamtai et al. 2022; Cheikh et al. 2016b; Boustany et al. 2023; Ezzikouri et al. 2008; Ezzikouri et al. 2011; Tazzite et al. 2012; Rahoui et al. 2014; Najdi et al. 2014; Cherif Chefchaouni et al. 2022).

## Discussion

To our knowledge, this is the first narrative review in Morocco to map the existing literature on real world data oncology studies. This synthesis of the 308 studies (1985–2025) reflects the progressive structuring of the national oncology research landscape. Research has evolved from single-center descriptive reports to registry-based cohorts, molecular studies, and health-system analyses. Output remains concentrated in some regions. The literature is dominated by breast, colorectal, and lung cancers, while other tumor types remain comparatively underrepresented. Since 2015, advances include wider use of molecular diagnostics, modernization of radiotherapy, and increasing attention to health-system challenges.

Yet, these national advances coexist with persistent structural limitations that mirror challenges seen across North Africa and other low- and middle-income countries (LMICs). While Morocco has taken meaningful steps toward RWD integration, the region as a whole continues to lag behind high-income settings where nationwide, interoperable databases and standardized reporting are routine (Jayathissa and Hewapathirana 2023; Bagherian and Sattari 2022). Within Morocco, the absence of a unified national oncology registry and the limited digitization of hospital records constrain data accessibility and continuity (Obtel et al. 2015). Existing regional registries in Casablanca and Rabatoperate intermittently and incompletely, reflecting broader infrastructural and governance gaps typical of LMICs in transition (Tazi et al. 2013b; Bouchbika et al. 2013b; Baladi et al. 2025).

Beyond these structural limitations, several pressing issues in the Moroccan cancer landscape remain insufficiently explored in the literature, including persistent geographic disparities in access to oncology services, delays in diagnosis and referral pathways, and the uneven distribution of oncology infrastructure and specialized workforce across regions (Selmouni et al. 2018). These challenges, highlighted in national cancer control evaluations, suggest that future research should prioritize implementation science approaches focusing on prevention, awareness, screening participation, and continuity of care from early detection to survivorship (Selmouni et al. 2018). Moreover, domains such as survivorship outcomes, long-term toxicity, psychosocial burden, and the economic impact of cancer care remain underrepresented in Moroccan real-world oncology studies despite their importance for informing policy and resource allocation (Selmouni et al. 2018).

Thus, Morocco stands as both a regional leader in oncology RWD generation and a representative of the systemic hurdles that still impede the full realization of data-driven cancer care across comparable contexts: Tunisia, Kenya, and Egypt for instance suggest that similar determinants-socio-economic disparities, centralization of care, and suboptimal data continuity-contribute to these outcomes (Ilinca et al. 2019; Giusti and Persiani 2024). Within the MENA region, few structured initiatives exist outside of the Saudi Cancer Registry and the National Cancer Network of Egypt (NCC-Egypt) (Saudi Cancer Registry, s. d.; Ibrahim et al. 2014). Strengthening screening programs, referral pathways, and survivorship monitoring could markedly improve cancer control indicators nationwide.

In contrast, high-income countries have developed robust ecosystems for RWD and RWE generation (Dang 2023). France, through the Institut National du Cancer (INCa) and the Système National des Données de Santé (SNDS), has established national data repositories and multicentric RWD programs such as ESMÉ, UNICANCER, and Epi-Roche (Bégaud et al. 2017). The United States has fully integrated RWD into its regulatory landscape; the FDA now accepts RWE in accelerated approval processes, supported by platforms such as Flatiron Health and SEER (Commissioner 2025).

In this context, Morocco occupies a strategic intermediary position between Europe and Africa. Several emerging initiatives-such as those led by the Mohammed VI center for research and innovation of Rabat (CM6RI), the National Institute of Oncology (INO), the Lalla Salma Foundation, and the cancer research institute of Fez-could serve as foundational pillars for a national RWD framework.

The barriers hindering RWD development in Morocco can be grouped into three categories: Structural barriers: absence of a comprehensive regulatory framework governing data privacy, sharing, and anonymization; lack of interoperability among hospital software systems (e.g., Sihati, Oasis); and limited digitalization of patient records in many university hospitals. Organizational barriers: the absence of an up-to-date national cancer registry, limited coordination between regional ethics committees for data research, and the lack of clinicians trained in data science and biostatistics. Cultural and economic barriers: a limited culture of data sharing and publication, institutional ownership of patient information, insufficient academic incentives to generate RWE, and lack of sustainable funding mechanisms for real-world studies.

Despite these obstacles, Morocco has a unique opportunity to leverage its institutional and human capital to structure RWD generation in oncology. Priority actions include: Integrating RWD objectives into the National Cancer Control Plan (2025–2030). Promoting public-private partnerships between academia, regulatory agencies, and the pharmaceutical industry for large-scale RWE programs is also recommended. Accelerating the digitalization of patient records through a unified Electronic Medical Record (EMR) system. Building capacity among oncologists, pharmacists, biostatisticians, and health-data scientists through targeted training and certification programs. Developing a clear ethical and legal framework for secondary data use and cross-institutional sharing. Engaging Morocco in international RWE networks, such as the Real-World Evidence Africa Network or the ESMO RWE Working Group.

The development of RWD in oncology extends beyond academic interest; it represents a crucial instrument for national health policy. Properly structured, RWD can serve as a decision-making tool for evaluating the cost-effectiveness, accessibility, and safety of new cancer therapies in real clinical settings. It also provides an evidence base for health-technology assessment, informs resource allocation, and supports regulatory and reimbursement decisions by bodies such as the ANAM and the AMO.

This review has inherent limitations. The available sources are incomplete, with many non-indexed, unpublished or inaccessible studies and limited methodological transparency. The analysis is likely restricted to published literature, excluding “grey” data such as theses, institutional reports, or conference abstracts. Further qualitative investigations-through stakeholder interviews and institutional case studies-are required to better understand the operational barriers to RWD generation.

In conclusion: cancer remains a major public health challenge in Morocco, and strengthening the use of RWD will be essential to guide future oncology policy and practice. Our synthesis of 308 Moroccan studies over four decades illustrates the country’s transition from isolated hospital case series to increasingly structured epidemiologic, molecular, and health-system research following the expansion of national cancer control initiatives. Despite these advances, important gaps persist, including the geographic concentration of research activity, fragmented data systems, and limited interoperability between registries and clinical datasets. Addressing these limitations through improved data integration, digitalization, and collaborative research networks will be critical. With sustained institutional commitment and strategic coordination, Morocco has the potential to consolidate its real-world oncology data ecosystem and serve as a model for strengthening evidence-based cancer control in comparable low- and middle-income countries.

## Supplementary Information

Below is the link to the electronic supplementary material.

Supplementary Material 1

## Data Availability

All data has been included in the main article and supplemental file.
