# Supplementary material for: Real-world data on oncology in Morocco: a narrative review of four decades of national evidence
Source: J Cancer Res Clin Oncol. 2026 May 13;152(7):140. doi: 10.1007/s00432-026-06460-6 (PMC13376321; doi:10.1007/s00432-026-06460-6)
Supplement: Supplementary file 1 — Supplementary Material 1 [file 432_2026_6460_MOESM1_ESM.docx]

**Supplementary data**

**Table 1 : search strategy**

| **Pubmed** | ((( case control studies[MeSH Terms] OR case control study[MeSH Terms] OR longitudinal studies[MeSH Terms] OR longitudinal study[MeSH Terms] OR retrospective studies[MeSH Terms] OR retrospective study[MeSH Terms] OR cohort study[MeSH Terms] OR cohort studies[MeSH Terms] OR cohort stud*[Title/Abstract] OR case-control stud*[Title/Abstract] OR follow up stud*[Title/Abstract] OR observational stud*[Title/Abstract] OR epidemiologic stud*[Title/Abstract] OR real-world OR real world OR real life  OR real-life OR RWD OR RWE OR real-world data OR real-world evidence ) **AND** ( cancer OR cancers OR cancerization OR cancerous OR onco* OR carcinoma* OR malignan* ) **AND** ( morocco[MeSH Terms] OR maroc*[Title/Abstract] OR morocc*[Title/Abstract] ))) NOT (case report[Title/Abstract] OR case reports[Title/Abstract] OR systematic review[Title/Abstract] OR systematic reviews[Title/Abstract] OR case-series[Title/Abstract]) |
| --- | --- |

**Terms used in combinations** : ((Group A) AND (Group B) AND (Group C)) NOT

(Group D) :

**Group A:**

- Mesh terms : case control studies/case control study/longitudinal studies/longitudinal study/retrospective studies/retrospective study/cohort study/cohort studies

- Title/abstract : cohort stud*/case-control stud*/follow up stud*/observational stud*/epidemiologic stud*

- All ﬁelds : real-world /real world / real life / real-life / RWD / RWE / real-world data / real-world evidence

**Group B :**

- All ﬁelds : cancer /cancers / cancerization / cancerous / onco* / carcinoma* / malignan*

**Group C :**

- Mesh terms : morocco

- Title / abstract : maroc*/morocc*

**Group D :**

- Title/ abstract : case report/case reports/systematic review/systematic reviews/ case-series

**References of the included 308 articles from the narrative review:**

Aazzane, O., Bakhtaoui, F. Z., Stitou, S., Fellah, H., & Karkouri, M. (2024). Clinicopathological characteristics and tumor infiltrating immune cells associations of PD-L1 tumor expression in non-small cell lung cancer patients. *La Tunisie Médicale*, *102*(4). https://doi.org/10.62438/tunismed.v102i4.4688

Aazzane, O., Fathi, S., Charkaoui, M., Acharki, A., Sahraoui, S., Benchakroun, N., Fellah, H., & Karkouri, M. (2024). Immunotherapy and PD-L1 Tumor Expression in Moroccan Non-Small Cell Lung Cancer Patients with Various Metastasis. *Asian Pacific Journal of Cancer Prevention*, *25*(8), 2841‑2852. https://doi.org/10.31557/APJCP.2024.25.8.2841

Aazzane, O., Mellouki, A., Fathi, S., Charkaoui, M., Stitou, S., Acharki, A., Benchakroun, N., Sahraoui, S., Fellah, H., & Karkouri, M. (2025). The Predictive Significance of Galectin 3 Expression in Patients with Unresectable Non-Small Cell Lung Cancer without EGFR or ALK Mutations Treated Platinum-Based Doublet Chemotherapy. *Asian Pacific Journal of Cancer Prevention*, *26*(4), 1407‑1419. https://doi.org/10.31557/APJCP.2025.26.4.1407

Abbass, F., Bennis, S., Znati, K., Akasbi, Y., Amrani, J. K., El Mesbahi, O., & Amarti, A. (2011). Le profil épidémiologique et biologique du cancer du sein à Fès-Boulemane (Maroc). *Eastern Mediterranean Health Journal*, *17*(11), 930‑936. https://doi.org/10.26719/2011.17.12.930

Abdou, J., Elkabous, M., M’rabti, H., & Errihani, H. (2015). Les sarcomes des tissus mous : À propos de 33 cas. *Pan African Medical Journal*, *22*. https://doi.org/10.11604/pamj.2015.22.374.8391

Abounouh, K., Aitraise, I., Benabou, A., Boussakri, I., Doumir, M. A., El Boussairi, C., El Idrissi, S., El Mahdaoui, C., Qouar, D. E., Ennahal, A., Fathi, S., Hafidi, M., Lachker, L., Ratib, C., Tanouti, I.-A., Maaroufi, A., Benjelloun, S., Guessous, F., Pineau, P., & Ezzikouri, S. (2019). Virus-associated human cancers in Moroccan population : From epidemiology to prospective research. *Infection, Genetics and Evolution*, *75*, 103990. https://doi.org/10.1016/j.meegid.2019.103990

Abrini, H., Amzerin, M., El Baaboua, A., Aboulaghras, S., Bouhda, A., & El Mrabet, F. Z. (2023). Comparison of different Pancreatic cancer treatments : A three-year retrospective study in the oncology center of Tangier university hospital, Morocco. *BMC Gastroenterology*, *23*(1), 452. https://doi.org/10.1186/s12876-023-03071-0

Ahanidi, H. E., Azzouzi, M. E., Arrouchi, H., Alaoui, C. H., Tetou, M., Bensaid, M., Oukabli, M., Ameur, A., Bouzidi, A. A., Mzibri, M. E., & Attaleb, M. (s. d.). *AKT1 and PIK3CA activating mutations in Moroccan bladder cancer patients´ biopsies and matched urine*.

Ainahi, A., Kebbou, M., Timinouni, M., Benabdeljalil, N., & Oufara, S. (2006). Treatment evaluation, follow-up and familial screening of medullary thyroid carcinoma by highly specific calcitonin measurements. *Indian Journal of Cancer*, *43*(2), 75. https://doi.org/10.4103/0019-509X.25888

Ait Boujmia, O. K., Nadifi, S., Dehbi, H., Lamchahab, M., & Quessar, A. (2020a). Association of Multidrug Resistance Gene-1 (MDR1 C1236T) Polymorphism with the Risk of Acute Myeloid Leukemia in a Moroccan Population. *Asian Pacific Journal of Cancer Prevention*, *21*(7), 1899‑1904. https://doi.org/10.31557/APJCP.2020.21.7.1899

Ait Boujmia, O. K., Nadifi, S., Dehbi, H., Lamchahab, M., & Quessar, A. (2020b). The influence of DNMT3A and DNMT3B gene polymorphisms on acute myeloid leukemia risk in a Moroccan population. *Current Research in Translational Medicine*, *68*(4), 191‑195. https://doi.org/10.1016/j.retram.2020.08.001

Aitelhaj, M., LKhouyaali, S., Rais, G., Mohtaram, A., Raissouni, S., Ghissassi, B., Boutayeb, S., Mrabti, H., Bensouda, Y., & Errihani, H. (2013). Cardiac safety of the adjuvant Trastuzumab in a Moroccan population : Observational monocentric study of about 100 patients. *BMC Research Notes*, *6*(1), 339. https://doi.org/10.1186/1756-0500-6-339

Aitelhaj, M., LKhoyaali, S., Rais, G., Boutayeb, S., & Errihani, H. (2016). First line chemotherapy plus trastuzumab in metastatic breast cancer HER2 positive—Observational institutional study. *Pan African Medical Journal*, *24*. https://doi.org/10.11604/pamj.2016.24.324.4058

Akhouayri, L., Regragui, M., Benayed, S., Guebessi, N. B., Marnissi, F., Chiorino, G., & Karkouri, M. (2022). Ki-67 proliferation index to further stratify invasive breast cancer molecular subtypes : Northern African comparative cohort-study with external TCGA-BRCA and metabric validation. *Pan African Medical Journal*, *41*. https://doi.org/10.11604/pamj.2022.41.170.31239

Akil, A., Ezzikouri, S., El Feydi, A. E., Benazzouz, M., Afifi, R., Diagne, A. G., Benjouad, A., Dejean, A., Pineau, P., & Benjelloun, S. (2012). Associations of genetic variants in the transcriptional coactivators EP300 and PCAF with hepatocellular carcinoma. *Cancer Epidemiology*, *36*(5), e300‑e305. https://doi.org/10.1016/j.canep.2012.05.011

Al Jarroudi, O., Zaimi, A., Brahmi, S. A., & Afqir, S. (2019). Nottingham Prognostic Index is an Applicable Prognostic Tool in Non-Metastatic Triple-Negative Breast Cancer. *Asian Pacific Journal of Cancer Prevention*, *20*(1), 59‑63. https://doi.org/10.31557/APJCP.2019.20.1.59

Alaoui Slimani, K., Debbagh, A., Sbitti, Y., Errihani, H., & Ichou, M. (2016). Cancer du sein chez l’homme au Maroc : Épidémiologie et facteurs pronostiques. À propos de 140 cas. *Gynécologie Obstétrique & Fertilité*, *44*(11), 636‑640. https://doi.org/10.1016/j.gyobfe.2016.08.009

Allali, M., Errafii, K., Fermi, R. E., Messaoudi, N., Fichtali, K., Fazazi, H. E., Ghanmi, A. E., Majjaoui, S. E., Ismaili, N., Wakrim, L., Idrissi, N. A., Rhalem, A. W., Ghazi, B., Ouladlahsen, A., Bakri, Y., Ghazal, H., & Hamdi, S. (2025). Risk factors for cervical cancer in Morocco : A case-control study. *Pan African Medical Journal*, *50*. https://doi.org/10.11604/pamj.2025.50.87.45024

Allam, L., Arrouchi, H., Ghrifi, F., El Khazraji, A., Kandoussi, I., Bendahou, M. A., El Amri, H., El Absi, M., & Ibrahimi, A. (2020). AKT1 Polymorphism (rs10138227) and Risk of Colorectal Cancer in Moroccan Population : A Case Control Study. *Asian Pacific Journal of Cancer Prevention*, *21*(11), 3165‑3170. https://doi.org/10.31557/APJCP.2020.21.11.3165

Aloulou, S., Mahfoudi, A. E., Omrani, A. E., & Khouchani, M. (2015). Facteurs liés au diagnostic tardif du cancer du sein : Expérience du CHU Mohammed VI Marrakech. *Pan African Medical Journal*, *21*. https://doi.org/10.11604/pamj.2015.21.162.4363

Ammor, A., Kisra, M., Oulahyane, R., Kababri, M., Maalmi, N., Cherkaoui, A., Bouhafs, A., Kaddouri, N., Abdelhak, M., Khattab, M., Alhamany, Z., & Benhmamouch, M. (2012). Ovarian tumours in children : A review of 18 cases. *African Journal of Paediatric Surgery*, *9*(3), 231. https://doi.org/10.4103/0189-6725.104726

Ammor, Y. M., Kaitouni, Z. I., Darfaoui, M., Lalya, I., Elomrani, A., & Khouchani, M. (2020). Managing cancer patients during COVID-19 pandemic : A North African Oncological center experience. *Pan African Medical Journal*, *35*. https://doi.org/10.11604/pamj.supp.2020.35.2.24582

Amrani Hassani Joutei, H., Mahfoud, W., Sadaoui, I., Fechtali, T., & Benomar, H. (2020). Étude des caractéristiques épidémiologiques cliniques et anatomopathologiques de l’adénocarcinome gastrique chez une population Marocaine. *Annales de Pathologie*, *40*(6), 442‑446. https://doi.org/10.1016/j.annpat.2020.04.014

Amri, F., Koulali, H., Jabi, R., Zazour, A., Bouziane, M., Ismaili, Z., & Kharrasse, G. (2023). Pancreatic cancer : Experience from an emerging country in North Africa. *Journal of Cancer Research and Clinical Oncology*, *149*(15), 14297‑14302. https://doi.org/10.1007/s00432-023-05245-5

Araqi Houssaini, L., Hali, F., Quessar, A., Marnissi, F., & Chiheb, S. (2021). Complications cutanées post-allogreffe de cellules souches hématopoïétiques. *Bulletin du Cancer*, *108*(9), 877‑885. https://doi.org/10.1016/j.bulcan.2021.03.015

Arechkik, A., El Hatimi, M., Amehmoud, H., Mahlaq, S., Blaak, H., Abdelkodouss Adidi, S., Lahlou, L., Obtel, M., & Razine, R. (2024). Health Related Quality of Life and Religiosity of Women with Cervical Cancer in the Souss-Massa Region, Morocco : A Cross-Sectional Study. *Asian Pacific Journal of Cancer Prevention*, *25*(12), 4351‑4358. https://doi.org/10.31557/APJCP.2024.25.12.4351

Ayoubi, S. E., Elkarroumi, M., El Khachibi, M., Hassani Idrissi, H., Ayoubi, H., Ennachit, S., Arazzakou, M., & Nadifi, S. (2018). The 72Pro Variant of the Tumor Protein 53 Is Associated with an Increased Breast Cancer Risk in the Moroccan Population. *Pathobiology*, *85*(4), 247‑253. https://doi.org/10.1159/000489852

Aznag, F. Z., Elouilamine, E., Korrida, A., & Izaabel, E. H. (2020). Polymorphisms in the Tumor Necrosis Factor Genes Are Associated with Breast Cancer in the Moroccan Population. *Genetic Testing and Molecular Biomarkers*, *24*(9), 592‑599. https://doi.org/10.1089/gtmb.2020.0073

Baghad, I., Erreguibi, D., Boufettal, R., Eljai, S. R., Chihab, F., & Nadifi, S. (2021). Association du polymorphisme de la méthylènetétrahydrofolate réductase C677T avec le risque de cancer colorectal sporadique. *Pan African Medical Journal*, *38*. https://doi.org/10.11604/pamj.2021.38.287.12522

Bahri, O., Ezzikouri, S., Alaya-Bouafif, N. B., Iguer, F., Feydi, A. E. E., Mestiri, H., Benazzouz, M., Khalfallah, T., Afifi, R., Elkihal, L., Berkane, S., Marchio, A., Debzi, N., Dejean, A., Pineau, P., Triki, H., & Benjelloun, S. (2011). First multicenter study for risk factors for hepatocellular carcinoma development in North Africa. *World Journal of Hepatology*, *3*(1), 24. https://doi.org/10.4254/wjh.v3.i1.24

Bakkach, J., Mansouri, M., Derkaoui, T., Loudiyi, A., Fihri, M., Hassani, S., Barakat, A., Ghailani Nourouti, N., & Bennani Mechita, M. (2017). Clinicopathologic and prognostic features of breast cancer in young women : A series from North of Morocco. *BMC Women’s Health*, *17*(1), 106. https://doi.org/10.1186/s12905-017-0456-1

Barkat, A., Laamiri Fs, Otmani, & Ahid S. (2013). Lipid profile among Moroccan overweight women and breast cancer : A case-control study. *International Journal of General Medicine*, 439. https://doi.org/10.2147/IJGM.S40826

Basraoui, D., Jaafari, F., & Jalal, H. (2018). Imagerie des tumeurs orbitaires chez l’enfant. *Pan African Medical Journal*, *29*. https://doi.org/10.11604/pamj.2018.29.190.14671

Batlamous, B., Lkhoyaali, S., Omri, L., Nguema-Mipaka, M.-G.-F., Khalis, M., Inrhaoun, H., Naciri, S., El Ghissassi, I., Mrabti, H., Boutayeb, S., & Errihani, H. (2025). Clinical Characteristics of Toxicities of Immune Checkpoint Inhibitors and Their Impact on Efficacy in Solid Cancers : An Analysis of Real-World Data in Moroccan Patients. *JCO Global Oncology*, (11), e2400312. https://doi.org/10.1200/GO-24-00312

Belbaraka, R., Lalya, I., Boulaamane, L., Tazi, M., Benjaafar, N., Errihani, H., & Ayyad, U. C. (2013). Les facteurs de risque alimentaires du carcinome indifférencié du nasopharynx : Une étude cas témoin. *LA TUNISIE MEDICALE*, *91*.

Belghali, M. Y., Ba-M’hamed, S., Admou, B., Brahimi, M., & Khouchani, M. (2021). Caractéristiques épidémiologiques, cliniques, thérapeutiques et évolutives des patients atteints de glioblastome cérébral : Série de cas pris en charge au centre d´oncohématologie du Centre Hospitalier Universitaire Mohammed VI de Marrakech en 2016 et 2017. *Pan African Medical Journal*, *39*. https://doi.org/10.11604/pamj.2021.39.191.28298

Belhamidi, M. S., Sinaa, M., Kaoukabi, A., Krimou, H., Menfaa, M., Sakit, F., & Choho, A. (2018). Profil épidémiologique et anatomopathologique du cancer colorectal : À propos de 36 cas. *Pan African Medical Journal*, *30*. https://doi.org/10.11604/pamj.2018.30.159.15061

Bellaoui, N., Lahsoune, M., Nourichafi, N., Majd, A., Mifdal, H., & Benchemsi, N. (2010). Greffe de cellules souches hématopoïétiques au Maroc : À propos de 87 cas. *Transfusion Clinique et Biologique*, *17*(2), 63‑65. https://doi.org/10.1016/j.tracli.2010.03.001

Belmokhtar, K. Y., Tajir, M., Boulouiz, R., Bennani, A., Brahmi, S. A., Alloubi, I., Kouismi, H., Kamaoui, I., Skiker, I., Afqir, S., Abda, N., Bellaoui, M., & Mezouar, L. (2019). Cancer du poumon au Maroc Oriental : Où en sommes-nous? *Pan African Medical Journal*, *34*. https://doi.org/10.11604/pamj.2019.34.177.19934

Benbakh, M., Abou-elfadl, M., Rouadi, S., Abada, R.-L., Roubal, M., & Mahtar, M. (2016). Substernal goiter : Experience with 50 cases. *European Annals of Otorhinolaryngology, Head and Neck Diseases*, *133*(1), 19‑22. https://doi.org/10.1016/j.anorl.2015.09.007

Benbrahim, Z., Berrada, A., Amaadour, L., Zahra El M’rabet, F., Elfatemi, H., Elfakir, S., Mellas, N., & Arifi, S. (2017). Étude comparative du cancer du sein localement avancé inflammatoire et non inflammatoire : Expérience d’un centre hospitalier marocain. *Gynécologie Obstétrique Fertilité & Sénologie*, *45*(11), 604‑608. https://doi.org/10.1016/j.gofs.2017.09.005

Benider, A., Bendahhou, K., Sauvaget, C., Mrabti, H., Selmouni, F., Muwonge, R., Alaoui, L., Lucas, E., Chami, Y., Abousselham, L., Bennani, M., Errihani, H., Sankaranarayanan, R., Bekkali, R., & Basu, P. (2022). Evolution of patterns of care for women with cervical cancer in Morocco over a decade. *BMC Cancer*, *22*(1), 479. https://doi.org/10.1186/s12885-022-09358-x

Benlhachemi, S., Khattab, M., Hattoufi, K., Abouqal, R., Boutayeb, S., & El Fahime, E. (2024). Analysis of Wilms Tumour Epidemiology, Clinicopathological Features and Treatment Outcomes in 84 Moroccan Patients. *Cancer Reports*, *7*(11), e2158. https://doi.org/10.1002/cnr2.2158

Benlhachemi, S., Khattab, M., Hattoufi, K., Abouqal, R., & El Fahime, E. (2025). Impact of neoadjuvant chemotherapy on tumour volume in unilateral Wilms tumour histotypes : A retrospective study. *BMC Cancer*, *25*(1), 1031. https://doi.org/10.1186/s12885-025-14177-x

Benmalek, R., Krikez, I., Maaroufi, A., Habbal, R., Haffadi, M., Tawfiq, N., & Benider, A. (s. d.). *Evaluation de la cardiotoxicité des traitements systémiques à l’unité de cardio-oncologie du CHU de Casablanca : Etude observationnelle prospective à propos de 1092 cas.*

Bennis, S., Abbass, F., Akasbi, Y., Znati, K., Joutei, K. A., El Mesbahi, O., & Amarti, A. (2012). Prevalence of molecular subtypes and prognosis of invasive breast cancer in north-east of Morocco : Retrospective study. *BMC Research Notes*, *5*(1), 436. https://doi.org/10.1186/1756-0500-5-436

Bennis, S., Meniar, S., Amarti, A., & Bijou, A. (2007). Role of cervical smear in the diagnosis of cervical cancer in Fes-Boulemane region of Morocco. *Eastern Mediterranean Health Journal*, *13*(5), 1153‑1159. https://doi.org/10.26719/2007.13.5.1153

Benyaich, Z., Hajhouji, F., Laghmari, M., Ghannane, H., Aniba, K., Lmejjati, M., & Ait Benali, S. (2020). Awake Craniotomy with Functional Mapping for Glioma Resection in a Limited-Resource-Setting : Preliminary Experience from a Lower-Middle Income Country. *World Neurosurgery*, *139*, 200‑207. https://doi.org/10.1016/j.wneu.2020.04.039

Berhili, S., Ouabdelmoumen, A., Sbai, A., Kebdani, T., Benjaafar, N., & Mezouar, L. (2019). Radical Mastectomy Increases Psychological Distress in Young Breast Cancer Patients : Results of A Cross-sectional Study. *Clinical Breast Cancer*, *19*(1), e160‑e165. https://doi.org/10.1016/j.clbc.2018.08.013

Berrada, S., Naim, S., Benayad, S., Benmoussa, A., Lamchahab, M., Rachid, M., Madani, A., & Qachouh, M. (2024). Real-World Efficacy and Toxicity of the LMBA02 Protocol for Burkitt Lymphoma in Morocco. *Clinical Lymphoma Myeloma and Leukemia*, *24*, S215. https://doi.org/10.1016/S2152-2650(24)00809-7

Berraho, M., Amarti-Riffi, A., El-Mzibri, M., Bezad, R., Benjaafar, N., Benideer, A., Matar, N., Qmichou, Z., Abda, N., Attaleb, M., Znati, K., El Fatemi, H., Bendahhou, K., Obtel, M., Filali Adib, A., Mathoulin-Pelissier, S., & Nejjari, C. (2017). HPV and cofactors for invasive cervical cancer in Morocco : A multicentre case-control study. *BMC Cancer*, *17*(1), 435. https://doi.org/10.1186/s12885-017-3425-z

Berraho, M., Bendahhou, K., Obtel, M., Zidouh, A., Benider, A., Errihani, H., & Nejjari, C. (2012). Cervical Cancer in Morocco : Epidemiological Profile from Two Main Oncological Centers. *Asian Pacific Journal of Cancer Prevention*, *13*(7), 3153‑3157. https://doi.org/10.7314/APJCP.2012.13.7.3153

Berthiller, J., Straif, K., Boniol, M., Voirin, N., Benhaïm-Luzon, V., Ayoub, W. B., Dari, I., Laouamri, S., Hamdi-Cherif, M., Bartal, M., Ayed, F. B., & Sasco, A. J. (2008). Cannabis Smoking and Risk of Lung Cancer in Men : A Pooled Analysis of Three Studies in Maghreb. *Journal of Thoracic Oncology*, *3*(12), 1398‑1403. https://doi.org/10.1097/JTO.0b013e31818ddcde

Bouaity, B., Darouassi, Y., Chihani, M., Touati, M. M., & Ammar, H. (2016). Analyse des facteurs prédictifs de malignité des goitres nodulaires : À propos de 500 cas. *Pan African Medical Journal*, *23*. https://doi.org/10.11604/pamj.2016.23.88.8405

Boufettal, H., Coullin, P., Mahdaoui, S., Noun, M., Hermas, S., & Samouh, N. (2011). Les môles hydatiformes complètes au Maroc : Étude épidémiologique et clinique. *Journal de Gynécologie Obstétrique et Biologie de la Reproduction*, *40*(5), 419‑429. https://doi.org/10.1016/j.jgyn.2011.02.008

Boufettal, H., & Samouh, N. (2015). Cancer du sein bilatéral synchrone au Maroc : Caractéristiques épidémiologiques et cliniques. *Pan African Medical Journal*, *20*. https://doi.org/10.11604/pamj.2015.20.118.6136

Boukansa, S., Benbrahim, Z., Gamrani, S., Bardai, S., Bouguenouch, L., Mazti, A., Boutahiri, N., Serraj, M., Amara, B., Ouadnouni, Y., Smahi, M., Alami, B., Mellas, N., & El Fatemi, H. (2022). Correlation of Epidermal Growth Factor Receptor Mutation With Major Histologic Subtype of Lung Adenocarcinoma According to IASLC/ATS/ERS Classification. *Cancer Control*, *29*, 10732748221084930. https://doi.org/10.1177/10732748221084930

Boukansa, S., Mouhrach, I., El Agy, F., El Bardai, S., Bouguenouch, L., Serraj, M., Amara, B., Ouadnouni, Y., Smahi, M., Alami, B., Mellas, N., Benbrahim, Z., & El Fatemi, H. (2024). Clinicopathological and prognostic implications of EGFR mutations subtypes in Moroccan non-small cell lung cancer patients : A first report. *PLOS ONE*, *19*(6), e0298721. https://doi.org/10.1371/journal.pone.0298721

Boukhris, S. A., Benajah, D. -a., Rhazi, K., Ibrahimi, S. A., Nejjari, C., Amarti, A., Mahmoud, M., Abkari, M., Souleimani, A., & Bennani, B. (2012). Prevalence and distribution of Helicobacter pylori cagA and vacA genotypes in the Moroccan population with gastric disease. *European Journal of Clinical Microbiology & Infectious Diseases*, *31*(8), 1775‑1781. https://doi.org/10.1007/s10096-011-1501-x

Boukhris, S. A., Khadir, M. E., Karim, S., Souho, T., Benajah, D.-A., Ibrahimi, S. A., Chbani, L., Abkari, M. E., & Bennani, B. (2025). Gastric Cancer and Associated Pathogens : Is There Any Association in the Moroccan Region? *Japanese Journal of Infectious Diseases*, *78*(2), 99‑105. https://doi.org/10.7883/yoken.JJID.2024.147

Boulaamane, L., Boutayeb, S., & Errihani, H. (2012). Bevacizumab based chemotherapy in first line treatment of HER2 negative metastatic breast cancer : Results of a Moroccan observational institutional study. *BMC Research Notes*, *5*(1), 162. https://doi.org/10.1186/1756-0500-5-162

Boulaamane, L., Essaadi, I., Lalya, I., M’rabti, H., & Errihani, H. (2011). Impact psychosocial du cancer sur les adolescents et les jeunes adultes marocains : Expérience de l’Institut national d’oncologie de Rabat. *Bulletin du Cancer*, *98*(9), 981‑988. https://doi.org/10.1684/bdc.2011.1427

Bounder, G., Jouimyi, M. R., Boura, H., Touati, E., Michel, V., Badre, W., Jouhadi, H., Kadi, M., Eljihad, M., Benomar, H., Kettani, A., Lebrazi, H., & Maachi, F. (2020). Associations of the -238(G/A) and -308(G/A) TNF-α Promoter Polymorphisms and TNF-α Serum Levels with the Susceptibility to Gastric Precancerous Lesions and Gastric Cancer Related to Helicobacter pylori Infection in a Moroccan Population. *Asian Pacific Journal of Cancer Prevention*, *21*(6), 1623‑1629. https://doi.org/10.31557/APJCP.2020.21.6.1623

Bourakkadi Idrissi, M., El Bouhaddouti, H., Mouaqit, O., Ousadden, A., Ait Taleb, K., & Benjelloun, E. B. (2023). Left-Sided Colon Cancer and Right-Sided Colon Cancer : Are They the Same Cancer or Two Different Entities? *Cureus*. https://doi.org/10.7759/cureus.37563

Bourhafour, M., Belbaraka, R., Souadka, A., M’rabti, H., Tijami, F., & Errihani, H. (2011a). Male breast cancer : A report of 127 cases at a Moroccan institution. *BMC Research Notes*, *4*(1), 219. https://doi.org/10.1186/1756-0500-4-219

Bourhafour, M., Belbaraka, R., Souadka, A., M’rabti, H., Tijami, F., & Errihani, H. (2011b). Male breast cancer : A report of 127 cases at a Moroccan institution. *BMC Research Notes*, *4*(1), 219. https://doi.org/10.1186/1756-0500-4-219

Boustany, Y., Laraqui, A., El Zaitouni, S., Ghaouti, M., Benzekri, A., Kettani, F., Oukabli, M., Ennibi, K., Belkadi, B., & Sekhsokh, Y. (2023). Advanced Non-small Cell Lung Cancer : EGFR Mutation Analysis Using Pyrosequencing and the Fully Automated qPCR-Based Idylla^TM^ System. *Cancer Control*, *30*, 10732748231177538. https://doi.org/10.1177/10732748231177538

Bouzini, G., Amzerin, M., Mahdi, Z., Sammoud, K., Chahbar, A., Najdi, A., & El M’Rabet, F. Z. (2025). Impact of the Basic Mandatory Health Insurance “AMO-Tadamon” on Continuity of Care Among Breast Cancer Patients Treated at the Oncology Center of the CHU Mohammed VI in Tangier : A Mixed Longitudinal Cohort Study. *Cancer Management and Research*, *Volume 17*, 851‑861. https://doi.org/10.2147/CMAR.S514238

Brahmi, S. A., Ziani, F. Z., Youssef, S., & Afqir, S. (2016). Aménorrhée chimio induite chez une population marocaine : À propos d’une cohorte rétrospective. *Pan African Medical Journal*, *24*. https://doi.org/10.11604/pamj.2016.24.58.8892

Chaouki, N., Bosch, F. X., Muñoz, N., Meijer, C. J. L. M., El Gueddari, B., El Ghazi, A., Deacon, J., Castellsagué, X., & Walboomers, J. M. M. (1998). The viral origin of cervical cancer in Rabat, Morocco. *International Journal of Cancer*, *75*(4), 546‑554. https://doi.org/10.1002/(SICI)1097-0215(19980209)75:4%253C546::AID-IJC9%253E3.0.CO;2-T

Chbani, L., Hafid, I., Berraho, M., Mesbahi, O., Nejjari, C., & Amarti, A. (2013). Epidemiological and pathological features of cancer in Fez Boulemane region, [Morocco]. *Eastern Mediterranean Health Journal*, *19*(03), 263‑270. https://doi.org/10.26719/2013.19.3.263

Chbani, L., Hafid, I., Berraho, M., Nejjari, C., & Amarti, A. (s. d.). *Digestive cancers in Morocco : Fez-Boulemane region*.

Chebak, M., Azzouzi, M., Chaibi, H., Fakhkhari, M., Benamri, I., Mguil, M., Hajjout, K., Zegmout, A., Tiresse, N., Rhorfi, I., Souhi, H., Abid, A., Zahraoui, R., Bourkadi, J., Oumzil, H., Radouani, F., & Sadki, K. (2023). Assessment of the Association of Chlamydia e pneumoniae Infection with Lung Cancer in a Moroccan Patients’ Cohort. *Asian Pacific Journal of Cancer Prevention*, *24*(2), 659‑665. https://doi.org/10.31557/APJCP.2023.24.2.659

Chebihi, Z. T., Belkhayat, A., Chadli, E., Hilal, L., Skhoun, H., Hessissen, L., El Khorassani, M., El Kababri, M., Kili, A., Khattab, M., Bakri, Y., & Dakka, N. (2018). Cytogenetic Profile of Moroccan Pediatric Acute Lymphoblastic Leukemia : Analysis of 155 Cases With a Review of the Literature. *Clinical Lymphoma Myeloma and Leukemia*, *18*(6), e241‑e248. https://doi.org/10.1016/j.clml.2018.04.004

Cheikh, A., Majjaoui, S. E., Ismaili, N., Cheikh, Z., Bouajaj, J., Nejjari, C., Hassani, A. E., Cherrah, Y., & Benjaafar, N. (2016). Evaluation of the cost of cervical cancer at the National Institute of Oncology, Rabat. *Pan African Medical Journal*, *23*. https://doi.org/10.11604/pamj.2016.23.209.7750

Chenna, H., Berhil, H., Nouni, K., Kabbaj, H., Zaidi, H., Toulba, A., Kacemi, H. E., Kebdani, T., Gueddari, B. K. E., & Benjaafar, N. (s. d.). *Le lymphomes non hodgkinien primitif de la thyroïde : À propos de sept cas*.

Cherif Chefchaouni, A., Moutaouakkil, Y., Adouani, B., Tadlaoui, Y., Lamsaouri, J., & Bousliman, Y. (2022). Impact of anti-cancer drugs shortages in oncology and hematology departments in a Moroccan hospital. *Journal of Oncology Pharmacy Practice*, *28*(4), 822‑826. https://doi.org/10.1177/10781552211008212

Cherkaoui, G. S., Guensi, A., Taleb, S., Idir, M. A., Touil, N., Benmoussa, R., Baroudi, Z., & Chikhaoui, N. (2015). Poorly differentiated thyroid carcinoma : A retrospective clinicopathological study. *Pan African Medical Journal*, *21*. https://doi.org/10.11604/pamj.2015.21.137.6720

Cherradi, I., Ichou, M., Houssaini, M. S., & Ismaili, N. (2025). Management of immune-related adverse events under PD-1/PD-L1 inhibitors : Insights from a Moroccan real-world experience. *Cancer Treatment and Research Communications*, *44*, 100978. https://doi.org/10.1016/j.ctarc.2025.100978

Chibani, H., El Bairi, K., Al Jarroudi, O., & Afqir, S. (2022). Bevacizumab in metastatic colorectal cancer in a real-life setting – toxicity profile, survival outcomes, and impact of tumor sidedness. *Współczesna Onkologia*, *26*(1), 32‑39. https://doi.org/10.5114/wo.2022.114678

Cissé, K., Adjadé, G., El Fadli, M., Essadi, I., & Belbaraka, R. (2024a). Factors linked to the late diagnosis of breast cancer and the initiation of treatment. *The Pan African Medical Journal*, *47*. https://doi.org/10.11604/pamj.2024.47.207.42734

Cissé, K., Adjadé, G., El Fadli, M., Essadi, I., & Belbaraka, R. (2024b). Factors linked to the late diagnosis of breast cancer and the initiation of treatment. *The Pan African Medical Journal*, *47*. https://doi.org/10.11604/pamj.2024.47.207.42734

Corsini, C., Henouda, S., Nejima, D. B., Bertet, H., Toledano, A., Boussen, H., Habib, F., Mouhout, A., Gaballah, A., Ghazaly, H. E., Bourgier, C., Coupier, I., Galibert, V., Baudry, K., Vilquin, P., Biquard, L., Rey, J.-M., Belkacemi, Y., Ihout, P., … Pujol, P. (2017). Early onset breast cancer : Differences in risk factors, tumor phenotype, and genotype between North African and South European women. *Breast Cancer Research and Treatment*, *166*(2), 631‑639. https://doi.org/10.1007/s10549-017-4434-y

Dahbi, Z., Elmejjatti, F., Naciri, F., Guerouaz, A., Oabdelmoumen, A., Sbai, A., & Mezouar, L. (2018). Les traitements du cancer de la vulve : Expérience du Centre d’Oncologie d’Oujda. *Pan African Medical Journal*, *31*. https://doi.org/10.11604/pamj.2018.31.182.13812

Dahbi, Z., Sbai, A., & Mezouar, L. (2018). Sexuality of Moroccan Survivors of Cervical Cancer : A Prospective Data. *Asian Pacific Journal of Cancer Prevention*, *19*(11), 3077‑3079. https://doi.org/10.31557/APJCP.2018.19.11.3077

Dakka, N., Bellaoui, H., Bouzid, N., Khattab, M., Bakri, Y., & Benjouad, A. (2009). CD10 AND CD34 EXPRESSION IN CHILDHOOD ACUTE LYMPHOBLASTIC LEUKEMIA IN MOROCCO : Clinical Relevance and Outcome. *Pediatric Hematology and Oncology*, *26*(4), 216‑231. https://doi.org/10.1080/07357900902897557

Dakkoune, M., Qachouh, M., Zoukal, S., Hassoune, S., Khoubila, N., Cherkaoui, S., Lamchahab, M., Rachid, M., Madani, A., & Quessar, A. (2020). Imatinib dans le traitement de la leucémie myéloide chronique au Maroc. *Bulletin Du Cancer*, *107*(9), 861‑866. https://doi.org/10.1016/j.bulcan.2020.05.013

Dardari, R., Khyatti, M., Cordeiro, P., Odda, M., ElGueddari, B., Hassar, M., & Menezes, J. (2006). High frequency of latent membrane protein‐1 30‐bp deletion variant with specific single mutations in Epstein‐Barr virus‐associated nasopharyngeal carcinoma in Moroccan patients. *International Journal of Cancer*, *118*(8), 1977‑1983. https://doi.org/10.1002/ijc.21595

Dardari, R., Khyatti, M., Jouhadi, H., Benider, A., Ettayebi, H., Kahlain, A., Mansouri, A., El Gueddari, B., & Benslimane, A. (2001). Study of human leukocyte antigen class I phenotypes in Moroccan patients with nasopharyngeal carcinoma. *International Journal of Cancer*, *92*(2), 294‑297. https://doi.org/10.1002/1097-0215(200102)9999:9999%253C::AID-IJC1177%253E3.0.CO;2-9

Darouassi, Y., Chihani, M., Touati, M. M., Ammar, H., & Bouaity, B. (2015). Apport diagnostique de la cervicotomie exploratrice : Étude rétrospective de 300 cas. *Pan African Medical Journal*, *22*. https://doi.org/10.11604/pamj.2015.22.364.8218

Darouassi, Y., Touati, M. M., Chihani, M., Alami, J. E., Bouaity, B., & Aamar, H. (2015). Les tumeurs malignes naso-sinusiennes : À propos de 32 cas et revues de la littérature. *Pan African Medical Journal*, *22*. https://doi.org/10.11604/pamj.2015.22.342.8220

DePasse, J., Caniza, M. A., Quessar, A., Khattab, M., Hessissen, L., Ribeiro, R., Cherkaoui, S., Benchekroun, S., & Matthay, K. K. (2013). Infections in hospitalized children and young adults with acute leukemia in Morocco. *Pediatric Blood & Cancer*, *60*(6), 916‑922. https://doi.org/10.1002/pbc.24365

Derkaoui, T., Bakkach, J., Mansouri, M., Loudiyi, A., Fihri, M., Alaoui, F. Z., Barakat, A., El Yemlahi, B., Bihri, H., Nourouti, N. G., & Mechita, M. B. (2016). Triple negative breast cancer in North of Morocco : Clinicopathologic and prognostic features. *BMC Women’s Health*, *16*(1), 68. https://doi.org/10.1186/s12905-016-0346-y

Diakité, A., Nouni, K., Bellefqih, S., Kebdani, T., & Benjaafar, N. (2014). Plasmocytome solitaire osseux : Expérience de l’institut national d’oncologie de Rabat (INO). *Pan African Medical Journal*, *17*. https://doi.org/10.11604/pamj.2014.17.180.2604

Diakite, B., Tazzite, A., Hamzi, K., Jouhadi, H., & Nadifi, S. (2012). Methylenetetrahydrofolate Reductase C677T polymorphism and breast cancer risk in Moroccan women. *African Health Sciences*, *12*(2), 204‑209. https://doi.org/10.4314/ahs.v12i2.20

Efared, B., Ebang, G. A., Tahiri, L., Sidibé, I. S., Erregad, F., Hammas, N., Melhouf, M. A., Banani, A., Chbani, L., & Fatemi, H. E. (2018). Phyllodes tumors of the breast : Clinicopathological analysis of 106 cases from a single institution. *Breast Disease*, *37*(3), 139‑145. https://doi.org/10.3233/BD-170297

El Agouri, H., Azizi, M., El Attar, H., El Khannoussi, M., Ibrahimi, A., Kabbaj, R., Kadiri, H., BekarSabein, S., EchCharif, S., Mounjid, C., & El Khannoussi, B. (2022). Assessment of deep learning algorithms to predict histopathological diagnosis of breast cancer : First Moroccan prospective study on a private dataset. *BMC Research Notes*, *15*(1), 66. https://doi.org/10.1186/s13104-022-05936-1

El Agy, F., El Bardai, S., Boukansa, S., Bouguenouch, L., Benbrahim, Z., Mazaz, K., Benjelloun, E. B., Ousadden, A., Ouldim, K., Ibrahimi, S. A., & Chbani, L. (2024). RAS Mutations Predict Recurrence-Free Survival and Recurrence Patterns in Colon Cancer : A Unicenter Study in Morocco. *Cancer Control*, *31*, 10732748241229290. https://doi.org/10.1177/10732748241229290

El Agy, F., El Bardai, S., El Otmani, I., Benbrahim, Z., Karim, I. M. H., Mazaz, K., Benjelloun, E. B., Ousadden, A., El Abkari, M., Ibrahimi, S. A., & Chbani, L. (2021). Mutation status and prognostic value of KRAS and NRAS mutations in Moroccan colon cancer patients : A first report. *PLOS ONE*, *16*(3), e0248522. https://doi.org/10.1371/journal.pone.0248522

El Alami, Y., Essangri, H., Majbar, M. A., Boutayeb, S., Benamr, S., El Malki, H. O., & Souadka, A. (2021). Psychometric validation of the Moroccan version of the EORTC QLQ-C30 in colorectal Cancer patients : Cross-sectional study and systematic literature review. *BMC Cancer*, *21*(1), 99. https://doi.org/10.1186/s12885-021-07793-w

El Badisy, I., BenBrahim, Z., Khalis, M., Elansari, S., ElHitmi, Y., Abbass, F., Mellas, N., & El Rhazi, K. (2024). Risk factors affecting patients survival with colorectal cancer in Morocco : Survival analysis using an interpretable machine learning approach. *Scientific Reports*, *14*(1), 3556. https://doi.org/10.1038/s41598-024-51304-3

El Bali, M., Mesmoudi, M., Essayah, A., Arbai, K., Ghailani Nourouti, N., Barakat, A., Sellal, N., & Bennani Mechita, M. (2024a). Epidemiological and anatomopathological profile of colorectal cancer in Northern Morocco between 2017 and 2019. *Arab Journal of Gastroenterology*, *25*(4), 338‑344. https://doi.org/10.1016/j.ajg.2024.10.002

El Bali, M., Mesmoudi, M., Essayah, A., Arbai, K., Ghailani Nourouti, N., Barakat, A., Sellal, N., & Bennani Mechita, M. (2024b). Epidemiological and anatomopathological profile of colorectal cancer in Northern Morocco between 2017 and 2019. *Arab Journal of Gastroenterology*, *25*(4), 338‑344. https://doi.org/10.1016/j.ajg.2024.10.002

El Fouhi, M., Benider, A., Kagambega Zoewendbem, A. G., & Mesfioui, A. (2020). Profil épidémiologique et anatomopathologique du cancer de sein au CHU Ibn Rochd, Casablanca. *Pan African Medical Journal*, *37*. https://doi.org/10.11604/pamj.2020.37.41.21336

El Hamdani, W., Hadami, K., Bensaid, M., El Ahanidi, H., Ameur, A., Filali Maltouf, A., Abbar, M., Attaleb, M., Albouzidi, A., & El Mzibri, M. (2017). Identification of G2607A mutation in EGFR gene with a significative rate in Moroccan patients with Bladder Cancer. *Cellular and Molecular Biology*, *63*(5), 75‑81. https://doi.org/10.14715/cmb/2017.63.5.14

El Hammoumi, M., El Oueriachi, F., Arsalane, A., & Kabiri, E. H. (2014). Surgical Management of Retrosternal Goitre : Experience of a Moroccan Center. *Acta Otorrinolaringologica (English Edition)*, *65*(3), 177‑182. https://doi.org/10.1016/j.otoeng.2014.05.001

El Kababi, S., Benajiba, M., El Khalfi, B., Hachim, J., & Soukri, A. (2019). Red blood cell alloimmunizations in beta-thalassemia patients in Casablanca/Morocco : Prevalence and risk factors. *Transfusion Clinique et Biologique*, *26*(4), 240‑248. https://doi.org/10.1016/j.tracli.2019.06.004

El Kababri, M., Benmiloud, S., Cherkaoui, S., El Houdzi, J., Maani, K., Ansari, N., Khoubila, N., Kili, A., El Khorassani, M., Madani, A., Tazi, M. A., Ahid, S., Hessissen, L., Quessar, A., Harif, M., Khattab, M., & André, N. (2020). Metro‐SMHOP 01 : Metronomics combination with cyclophosphamide‐etoposide and valproic acid for refractory and relapsing pediatric malignancies. *Pediatric Blood & Cancer*, *67*(9), e28508. https://doi.org/10.1002/pbc.28508

El Kinany, K., Hatime, Z., El Asri, A., Benslimane, A., Mint Sidi Deoula, M., Zarrouq, B., Lagiou, P., & El Rhazi, K. (2025). Adherence to the Mediterranean diet and colorectal cancer risk : A large case control study in the Moroccan population. *Public Health Nutrition*, *28*(1), e48. https://doi.org/10.1017/S1368980025000199

El Kinany, K., Huybrechts, I., Hatime, Z., El Asri, A., Boudouaya, H. A., Deoula, M. M. S., Kampman, E., & El Rhazi, K. (2022). Food processing groups and colorectal cancer risk in Morocco : Evidence from a nationally representative case–control study. *European Journal of Nutrition*, *61*(5), 2507‑2515. https://doi.org/10.1007/s00394-022-02820-3

El Kinany, K., Huybrechts, I., Kampman, E., Boudouaya, H. A., Hatime, Z., Mint Sidi Deoula, M., El Asri, A., Benslimane, A., Nejjari, C., Ibrahimi, S. A., Mrabti, H., Abda, N., Alaoui, R., Gunter, M. J., & El Rhazi, K. (2019). Concordance with the World Cancer Research Fund/American Institute for Cancer Research recommendations for cancer prevention and colorectal cancer risk in Morocco : A large, population‐based case–control study. *International Journal of Cancer*, *145*(7), 1829‑1837. https://doi.org/10.1002/ijc.32263

El Kinany, K., Mint Sidi Deoula, M., Hatime, Z., Boudouaya, H. A., Huybrechts, I., El Asri, A., Benider, A., Ahallat, M., Afqir, S., Mellas, N., Khouchani, M., & El Rhazi, K. (2020). Consumption of modern and traditional Moroccan dairy products and colorectal cancer risk : A large case control study. *European Journal of Nutrition*, *59*(3), 953‑963. https://doi.org/10.1007/s00394-019-01954-1

El Koubaiti, R., Mazti, A., Maaroufi, M., El Idrissi, M., El Ibrahimi, A., El Mrini, A., Bouhafa, T., El Fakir, S., Ouldim, K., Arifi, S., & Chbani, L. (2022). Molecular classification of soft tissue sarcomas for adequate diagnosis : A study on the northeast population of Morocco. *Heliyon*, *8*(9), e10673. https://doi.org/10.1016/j.heliyon.2022.e10673

El, M. R., Boudhas, A., Allaoui, M., Rharrassi, I., Chahdi, H., Bouzidi, A. A., & Oukabli, M. (2015). Les tumeurs annexielles cutanées : Étude anatomopathologique à propos de 96 cas. *Pan African Medical Journal*, *20*. https://doi.org/10.11604/pamj.2015.20.389.6202

El Maaroufi, H., Ababou, M., Hammani, A., Ahchouch, S., Jennane, S., Mahtat, M., Mikdmae, M., Messaoudi, N., & Doghmi, K. (2020). Prise en charge des syndromes myélodysplasiques au Maroc à propos d´une étude mono-centrique. *Pan African Medical Journal*, *37*. https://doi.org/10.11604/pamj.2020.37.300.20972

El Maataoui, A., Taoufiq, A., Fares, S., & Sokori, K. (2022). Étude transversale descriptive sur les profils cliniques et paracliniques des gammapathies monoclonales au niveau d´une région agricole du Souss-Massa au Maroc. *Pan African Medical Journal*, *41*. https://doi.org/10.11604/pamj.2022.41.69.32470

El Otmani, I., El Agy, F., El Abkari, M., Hassani, K. I. M., Mazaz, K., Benjelloun, E. B., Taleb, K. A., Bouhafa, T., Benbrahim, Z., Ibrahimi, S. A., & Chbani, L. (2020). The Effect of Lymph Nodes’ Histologic Response on Survival Outcomes in Moroccan Patients with Rectal Cancer. *International Journal of Surgical Oncology*, *2020*, 1‑7. https://doi.org/10.1155/2020/8406045

El Yaakoubi, A., Lahmadi, S., Benkabbou, A., Mohsine, R., Belkouchi, A., El Harroudi, T., El Malki, H. O., Hrora, A., Souadka, A., & Majbar, M. A. (2022). Audit of laparoscopic surgery for colon cancer in Morocco : A report of the results of a prospective multicentre cohort study. *Annals of Medicine & Surgery*, *80*. https://doi.org/10.1016/j.amsu.2022.104290

El Yacoubi, H., Sow, M. L., Kettani, F., Gamra, L., Mestari, A., Jabri, L., Elghissassi, I., & Errihani, H. (2020). Frequency of anaplastic lymphoma kinase rearrangements in Moroccan patients with non small cell lung cancer : A multi-institutional national retrospective study. *BMC Cancer*, *20*(1), 479. https://doi.org/10.1186/s12885-020-06973-4

El Zaitouni, S., Laraqui, A., Ghaouti, M., Benzekri, A., Kettani, F., Bajjou, T., Sekhsokh, Y., Benmokhtar, S., Jafari, M., Baba, W., Oukabli, M., El Annaz, H., Abi, R., Tagajdid, M. R., El Kochri, S., Lahlou, I. A., Ameziane El Hassani, R., & Ennibi, K. (2024). *KRAS* , *NRAS* and *BRAF* Mutational Profile of Colorectal Cancer in a Series of Moroccan Patients. *Cancer Control*, *31*, 10732748241262179. https://doi.org/10.1177/10732748241262179

El Zaitouni, S., Laraqui, A., Ghaouti, M., Benzekri, A., Kettani, F., Boustany, Y., Benmokhtar, S., Lamrani Alaoui, H., El Annaz, H., Abi, R., Tagajdid, M. R., El Kochri, S., El Mchichi, B., Bouaiti, E. A., Lahlou, I. A., El Hassani, R. A., & Ennibi, K. (2024). Genetic Profiling of Non-Small Cell Lung Cancer in Moroccan Patients by Targeted Next-Generation Sequencing. *Technology in Cancer Research & Treatment*, *23*, 15330338241288907. https://doi.org/10.1177/15330338241288907

Elalouani, C., Benhmidoun, M. A., Rida, H., AitRaiss, M., Derhem, N., Elomrani, A., Khouchani, M., Tahri, A., Errehmouni, A., Faouzi, R., Elguenzri, A., Elhattaoui, M., Tazi, I., & Mahmal, L. (2012). Cardiotoxicité à court et à moyen terme des anthracyclines : Étude prospective. *Annales de Cardiologie et d’Angéiologie*, *61*(4), 257‑266. https://doi.org/10.1016/j.ancard.2012.03.004

Elbachiri, M., Dao, A., Jabir, H., Sahraoui, S., Taleb, A., Bouchbika, Z., Benchakroun, N., Jouhadi, H., Tawfiq, N., & Benider, A. (2015). Résultats du traitement du médulloblastome à Casablanca de 2000 à 2012. *Cancer/Radiothérapie*, *19*(8), 718‑724. https://doi.org/10.1016/j.canrad.2015.05.026

Elbaylek, H., & Ammor, S. (2024). Adherence to the Mediterranean Diet and Colorectal Cancer Risk Among Moroccan Population : Hospital-Based Case Control Study. *Asian Pacific Journal of Cancer Prevention*, *25*(8), 2853‑2860. https://doi.org/10.31557/APJCP.2024.25.8.2853

Elbaylek, H., & Ammor, S. (2025). Oral Contraceptives Use and Colorectal Cancer Risk Among Moroccan Women : A Case-Control Study. *Asian Pacific Journal of Cancer Prevention*, *26*(6), 2225‑2232. https://doi.org/10.31557/APJCP.2025.26.6.2225

Elidrissi Errahhali, M., Elidrissi Errahhali, M., Abda, N., & Bellaoui, M. (2016). Exploring Geographic Variability in Cancer Prevalence in Eastern Morocco : A Retrospective Study over Eight Years. *PLOS ONE*, *11*(3), e0151987. https://doi.org/10.1371/journal.pone.0151987

Elidrissi Errahhali, M., Elidrissi Errahhali, M., Boulouiz, R., Ouarzane, M., & Bellaoui, M. (2016). Distribution and features of hematological malignancies in Eastern Morocco : A retrospective multicenter study over 5 years. *BMC Cancer*, *16*(1), 159. https://doi.org/10.1186/s12885-016-2205-5

Elidrissi Errahhali, M., Elidrissi Errahhali, M., Ouarzane, M., Boulouiz, R., & Bellaoui, M. (2017). Cancer incidence in eastern Morocco : Cancer patterns and incidence trends, 2005–2012. *BMC Cancer*, *17*(1), 587. https://doi.org/10.1186/s12885-017-3597-6

Elidrissi Errahhali, M., Elidrissi Errahhali, M., Ouarzane, M., El Harroudi, T., Afqir, S., & Bellaoui, M. (2017). First report on molecular breast cancer subtypes and their clinico-pathological characteristics in Eastern Morocco : Series of 2260 cases. *BMC Women’s Health*, *17*(1), 3. https://doi.org/10.1186/s12905-016-0361-z

Elkhalloufi, F., Boutayeb, S., Mamouch, F., Rakibi, L., Elazzouzi, S., & Errihani, H. (2021). The evolution of the socio-cultural and religious characteristics of cancer patients in Morocco : Case of the National Institute of Oncology Rabat. *BMC Cancer*, *21*(1), 516. https://doi.org/10.1186/s12885-021-08175-y

Elmajjaoui, S., Ismaili, N., El Kacemi, H., Kebdani, T., Sifat, H., & Benjaafar, N. (2016). Epidemiology and outcome of cervical cancer in national institute of Morocco. *BMC Women’s Health*, *16*(1), 62. https://doi.org/10.1186/s12905-016-0342-2

El Mansouri, M., Essaddouki, S., Mouradi, M., Oukerroum, A., El Fatoiki, F. Z., Truchuelo, M. T., Vitale, M. A., González, S., & Chiheb, S. (2023). Evaluation of the effectiveness and safety of combined oral and topical photoprotection with a standardized extract of *Polypodium leucotomos* (Fernblock®) in a Moroccan population with xeroderma pigmentosum. *Photodermatology, Photoimmunology & Photomedicine*, *39*(6), 607‑612. https://doi.org/10.1111/phpp.12904

Erefai, O., Soulaymani, A., Mokhtari, A., & Hami, H. (2022). Clinical and histopathological pattern of lung cancer in Morocco. *Pan African Medical Journal*, *42*. https://doi.org/10.11604/pamj.2022.42.283.35593

Errachdi, A., Asabbane, A., Nkoua Epala, B., Hemmich, M., Kabbali, N., Kebdani, T., & Benjaafar, N. (2014). Cancer avancé du col utérin : Aspects évolutif et pronostique. Expérience marocaine. *La Presse Médicale*, *43*(10), e257‑e264. https://doi.org/10.1016/j.lpm.2014.02.029

Erraji, H., Hali, F., Baline, K., Marnissi, F., & Chiheb, S. (2019). Profil épidémiologique, clinique et évolutif de 166 cas de mycosis fongoïde à Casablanca. *Annales de Dermatologie et de Vénéréologie*, *146*(12), 825‑828. https://doi.org/10.1016/j.annder.2019.08.006

Errihani, H., Berrada, N., Raissouni, S., Rais, F., Mrabti, H., & Rais, G. (2011). Classic Kaposi’s sarcoma in morocco : Clinico -epidemiological study at the national institute of oncology. *BMC Dermatology*, *11*(1), 15. https://doi.org/10.1186/1471-5945-11-15

Errihani, H., Mrabti, H., Boutayeb, S., El Ghissassi, I., El Mesbahi, O., Hammoudi, M., Chergui, H., & Riadi, A. (2008). Impact of cancer on Moslem patients in Morocco. *Psycho-Oncology*, *17*(1), 98‑100. https://doi.org/10.1002/pon.1200

Errihani, H., Mrabti, H., Sbitti, Y., Kaikani, W., El Ghissassi, I., Afqir, S., Boutayeb, S., Farik, M., Riadi, A., Hammoudi, M., & Chergui, H. (2010). Impact psychosocial et religieux du diagnostic de cancer chez les patients marocains : Expérience de l’Institut national d’oncologie de Rabat. *Bulletin du Cancer*, *97*(4), 461‑468. https://doi.org/10.1684/bdc.2010.1082

Essadik, A., Jouhadi, H., Rhouda, T., Nadifiyine, S., Kettani, A., & Maachi, F. (2015). Polymorphisms of Tumor Necrosis Factor Alpha in Moroccan Patients with Gastric Pathology : New Single‐Nucleotide Polymorphisms in TNF‐ *α*^−193^ (G/A). *Mediators of Inflammation*, *2015*(1), 143941. https://doi.org/10.1155/2015/143941

Ezzikouri, S., El Feydi, A. E., Afifi, R., Benazzouz, M., Hassar, M., Pineau, P., & Benjelloun, S. (2011). Impact of TP53 Codon 72 and MDM2 Promoter 309 Allelic Dosage in a Moroccan Population with Hepatocellular Carcinoma. *The International Journal of Biological Markers*, *26*(4), 229‑233. https://doi.org/10.5301/JBM.2011.8881

Ezzikouri, S., El Feydi, A. E., Afifi, R., El Kihal, L., Benazzouz, M., Hassar, M., Marchio, A., Pineau, P., & Benjelloun, S. (2009). MDM2 SNP309T>G polymorphism and risk of hepatocellular carcinoma : A case–control analysis in a Moroccan population. *Cancer Detection and Prevention*, *32*(5‑6), 380‑385. https://doi.org/10.1016/j.cdp.2009.01.003

Ezzikouri, S., El Feydi, A. E., El Kihal, L., Afifi, R., Benazzouz, M., Hassar, M., Chafik, A., Pineau, P., & Benjelloun, S. (2008). Prevalence of Common HFE and SERPINA1 Mutations in Patients with Hepatocellular Carcinoma in a Moroccan Population. *Archives of Medical Research*, *39*(2), 236‑241. https://doi.org/10.1016/j.arcmed.2007.09.006

Ezzikouri, S., Feydi, A. E. E., Afifi, R., Benazzouz, M., Hassar, M., Pineau, P., & Benjelloun, S. (2010). Polymorphisms in antioxidant defence genes and susceptibility to hepatocellular carcinoma in a Moroccan population. *Free Radical Research*, *44*(2), 208‑216. https://doi.org/10.3109/10715760903402906

Fakir, S. E., Najdi, A., Khazraji, Y. C., Bennani, M., Belakhel, L., Abousselham, L., Lyoussi, B., Bekkali, R., & Nejjari, C. (2015). Breast Cancer Screening in Morocco : Performance Indicators During Two Years of an Organized Programme. *Asian Pacific Journal of Cancer Prevention*, *16*(15), 6285‑6288. https://doi.org/10.7314/APJCP.2015.16.15.6285

Fares, S., Hadri, H., Rachid, M., Moutiqui, T., Oukkache, B., & Quessar, A. (s. d.). *Myélome multiple et autogreffe des cellules souches hématopoïétiques sans cryoconservation : Expérience du Service d´Hématologie Clinique de Casablanca au Maroc*.

Feng, B., Jalbout, M., Ayoub, W. B., Khyatti, M., Dahmoul, S., Ayad, M., Maachi, F., Bedadra, W., Abdoun, M., Mesli, S., Hamdi‐Cherif, M., Boualga, K., Bouaouina, N., Chouchane, L., Benider, A., Ben Ayed, F., Goldgar, D., & Corbex, M. (2007). Dietary risk factors for nasopharyngeal carcinoma in Maghrebian countries. *International Journal of Cancer*, *121*(7), 1550‑1555. https://doi.org/10.1002/ijc.22813

Feng, B.-J., Khyatti, M., Ben-Ayoub, W., Dahmoul, S., Ayad, M., Maachi, F., Bedadra, W., Abdoun, M., Mesli, S., Bakkali, H., Jalbout, M., Hamdi-Cherif, M., Boualga, K., Bouaouina, N., Chouchane, L., Benider, A., Ben-Ayed, F., Goldgar, D. E., & Corbex, M. (2009). Cannabis, tobacco and domestic fumes intake are associated with nasopharyngeal carcinoma in North Africa. *British Journal of Cancer*, *101*(7), 1207‑1212. https://doi.org/10.1038/sj.bjc.6605281

Fouad, A., Yousra, A., Kaoutar, Z., Omar, E. M., Afaf, A., & Sanae, B. (s. d.). *Classification moléculaire du cancer du sein au Maroc*.

Gallouj, S., Aqil, N., Harmouch, T., & Mernissi, F. Z. (2019). L’intérêt de la chirurgie micrographique dans la prise en charge du carcinome basocellulaire : Expérience du service de dermatologie CHU Hassan II de Fès, Maroc. *Pan African Medical Journal*, *33*. https://doi.org/10.11604/pamj.2019.33.245.18562

Ghouzlani, A., Lakhdar, A., Rafii, S., Karkouri, M., & Badou, A. (2021). The immune checkpoint VISTA exhibits high expression levels in human gliomas and associates with a poor prognosis. *Scientific Reports*, *11*(1), 21504. https://doi.org/10.1038/s41598-021-00835-0

Guennoun, A., Krimou, Y., Bouchikhi, C., Mamouni, N., Errarhay, S., & Banani, A. (2018). Corrélation radio-histologique des lésions mammaires ACR4 : À propos de 181 cas et revue de la littérature. *Pan African Medical Journal*, *29*. https://doi.org/10.11604/pamj.2018.29.140.13699

Gunden, J. R., Bendahhou, K., Benider, A., Ibrahim Khalil, A., Khalis, M., Wilson, M. L., & Soliman, A. S. (2019). Evaluating the incidence of inflammatory breast cancer using population- and hospital-based cancer registries in Casablanca, Morocco. *Breast Disease*, *38*(2), 39‑45. https://doi.org/10.3233/BD-180357

Hadadi, K., Hommadi, M., Belemlih, M., Zaghba, N., Maghous, A., Marnouch, E.-A., Saghir, K. A., Elmarjany, M., Sifat, H., Oukabli, M., & Mansouri, H. (2019). Plasmocytome solitaire osseux : Expérience du service de radiothérapie de l’hôpital militaire d’instruction Mohammed-V de Rabat (Maroc). *Cancer/Radiothérapie*, *23*(8), 867‑873. https://doi.org/10.1016/j.canrad.2019.06.013

Haddad, F., Nadir, S., Benkhaldoun, L., Alaoui, R., & Cherkaoui, A. (2005). Mélanome anorectal primitif. *La Presse Médicale*, *34*(2), 85‑88. https://doi.org/10.1016/S0755-4982(05)88233-8

Hajhouji, F., Lmejjati, M., Aniba, K., Laghmari, M., Ghannane, H., & Benali, S. A. (2017). Foramen magnum meningioma’s management : The experience of the department of neurosurgery in Marrakesh. *Pan African Medical Journal*, *26*. https://doi.org/10.11604/pamj.2017.26.42.10838

Hali, F., Latifi, A., & Sbai, M. (2011). Dégénérescence carcinomateuse des maux perforants plantaires d’origine lépreuse : Expérience du Centre national de léprologie de Casablanca. *Bulletin de la Société de pathologie exotique*, *104*(1), 6‑9. https://doi.org/10.1007/s13149-010-0126-4

Hardi, H., Melki, R., Boughaleb, Z., El Harroudi, T., Aissaoui, S., & Boukhatem, N. (2018). Significant association between ERCC2 and MTHR polymorphisms and breast cancer susceptibility in Moroccan population : Genotype and haplotype analysis in a case-control study. *BMC Cancer*, *18*(1), 292. https://doi.org/10.1186/s12885-018-4214-z

Harmouch, A., Taleb, M., Lasseini, A., Maher, M., & Sefiani, S. (2012). Epidemiology of pediatric primary tumors of the nervous system : A retrospective study of 633 cases from a single Moroccan institution. *Neurochirurgie*, *58*(1), 14‑18. https://doi.org/10.1016/j.neuchi.2012.01.005

Hassani, W., Alaoui, G. C., Soussy, K., Fatim-Zahra, F., Alami, Z., & Touria, B. (2025). Exploring therapeutic aspects and prognosis of Moroccan patients with vaginal cancer : A retrospective cohort study at the University Hospital of Fez. *Pan African Medical Journal*, *50*. https://doi.org/10.11604/pamj.2025.50.48.29059

Hassan-II, C. (2025). *Moncef Amrani Hassani Ahmed Filali Baba Meryem Alami Hazar Lahlou*. *20*.

Hatime, Z., El Kinany, K., Huybrechts, I., Gunter, M. J., Khalis, M., Deoula, M., Boudouaya, H. A., Benslimane, A., Nejjari, C., Benider, A., & El Rhazi, K. (2021). Extended healthy lifestyle index and colorectal cancer risk in the Moroccan population. *European Journal of Nutrition*, *60*(2), 1013‑1022. https://doi.org/10.1007/s00394-020-02311-3

Hatime, Z., El Kinany, K., Huybrechts, I., Murphy, N., Gunter, M. J., Khalis, M., Meimouna, S. D., Boudouaya, H. A., Benslimane, A., El Asri, A., Abkari, M., Bendahhou, K., Ismaili, M. Z., & El Rhazi, K. (2022). Association of Physical Activity and Sedentary Behavior with Colorectal Cancer Risk in Moroccan Adults : A Large-Scale, Population-Based Case–Control Study. *Asian Pacific Journal of Cancer Prevention*, *23*(6), 1859‑1866. https://doi.org/10.31557/APJCP.2022.23.6.1859

Hazmiri, F.-E., Boukis, F., Benali, S. A., El Ganouni, N. C. I., & Rais, H. (2018). Tumeurs cérébrales de l’enfant : À propos de 136 cas. *Pan African Medical Journal*, *30*. https://doi.org/10.11604/pamj.2018.30.291.13208

Hbid, O., Belloul, L., Fajali, N., Ismaili, N., Duprez, R., Tanguy, M., Benomar, H., Tahri, E. H., Gessain, A., & Huerre, M. (2005). Kaposi’s sarcoma in Morocco : A pathological study with immunostaining for human herpesvirus-8 LNA-1. *Pathology*, *37*(4), 288‑295. https://doi.org/10.1080/00313020500169453

Hessissen, L., Kanouni, L., Kili, A., Nachef, M. N., El Khorassani, M., Benjaafar, N., Khattab, M., & El Gueddari, B. E. K. (2010). Pediatric rhabdomyosarcoma in Morocco. *Pediatric Blood & Cancer*, *54*(1), 25‑28. https://doi.org/10.1002/pbc.22173

Hessissen, L., Khtar, R., Madani, A., El Kababri, M., Kili, A., Harif, M., Khattab, M., Sahraoui, S., Benjaafar, N., Ahid, S., Howard, S. C., & Benchekroun, S. (2013). Improving the prognosis of pediatric hodgkin lymphoma in developing countries : A moroccan society of pediatric hematology and oncology study: Pediatric Hodgkin Lymphoma in Morocco. *Pediatric Blood & Cancer*, *60*(9), 1464‑1469. https://doi.org/10.1002/pbc.24534

Houda, D., Ezzahra, I. F., Karima, B., Abdelatif, B., & Driss, R. (2020). Habitudes toxiques et comportements alimentaires de 305 cas du cancer du sein colligés au centre Mohammed VI pour les traitements des cancers de Casablanca. *Pan African Medical Journal*, *36*. https://doi.org/10.11604/pamj.2020.36.51.18869

Houjami, M., Sahraoui, S., Benchakroun, N., Jouhadi, H., Tawfiq, N., & Benider, A. (2011). Épendymomes intracrâniens : Étude rétrospective de 16 cas. *Cancer/Radiothérapie*, *15*(2), 136‑139. https://doi.org/10.1016/j.canrad.2010.09.001

Houssaini, K., Majbar, M. A., Souadka, A., Lahnaoui, O., El Ahmadi, B., Ghannam, A., Houssain Belkhadir, Z., Mohsine, R., & Benkabbou, A. (2022). Liver resection safety in a developing country : Analysis of a collective learning curve. *Journal of Visceral Surgery*, *159*(1), 5‑12. https://doi.org/10.1016/j.jviscsurg.2021.02.006

Imad, F. E., Drissi, H., Tawfiq, N., Bendahhou, K., Benider, A., & Radallah, D. (2020). Facteurs de risque alimentaires du cancer colorectal au Maroc : Étude cas témoin. *Pan African Medical Journal*, *35*. https://doi.org/10.11604/pamj.2020.35.59.18214

Imad, F. E., Drissi, H., Tawfiq, N., Bendahhou, K., Jouti, N. T., Benider, A., & Radallah, D. (2019a). Aspects épidémiologiques, nutritionnels et anatomopathologiques des cancers colorectaux dans la région du grand Casablanca. *Pan African Medical Journal*, *32*. https://doi.org/10.11604/pamj.2019.32.56.10548

Imad, F. E., Drissi, H., Tawfiq, N., Bendahhou, K., Jouti, N. T., Benider, A., & Radallah, D. (2019b). Influence des facteurs socio-économiques et du niveau d’éducation sur le cancer colorectal chez une population marocaine. *Pan African Medical Journal*, *34*. https://doi.org/10.11604/pamj.2019.34.209.18345

Issoufou, I., El Alami, H., Belliraj, L., Harmouchi, H., Ammor, F. Z., Lakranbi, M., Ouadnouni, Y., & Smahi, M. (2020). La chirurgie des tumeurs carcinoïdes trachéobronchiques : Bilan d’activité. *Revue des Maladies Respiratoires*, *37*(2), 117‑122. https://doi.org/10.1016/j.rmr.2019.08.007

Jadid, F. Z., Chihab, H., Alj, H. S., Elfihry, R., Zaidane, I., Tazi, S., Badre, W., Marchio, A., El Filali, K. M., Tahiri, M., Saile, R., Pineau, P., Ezzikouri, S., & Benjelloun, S. (2018a). Control of progression towards liver fibrosis and hepatocellular carcinoma by SOCS3 polymorphisms in chronic HCV-infected patients. *Infection, Genetics and Evolution*, *66*, 1‑8. https://doi.org/10.1016/j.meegid.2018.08.027

Jadid, F. Z., Chihab, H., Alj, H. S., Elfihry, R., Zaidane, I., Tazi, S., Badre, W., Marchio, A., El Filali, K. M., Tahiri, M., Saile, R., Pineau, P., Ezzikouri, S., & Benjelloun, S. (2018b). Control of progression towards liver fibrosis and hepatocellular carcinoma by SOCS3 polymorphisms in chronic HCV-infected patients. *Infection, Genetics and Evolution*, *66*, 1‑8. https://doi.org/10.1016/j.meegid.2018.08.027

Janane, A., Jawad, C., Hajji, F., Ould, T., Ghadouane, M., Ameur, A., Abbar, M., & Albouzidi, A. (2011). Resultados de gammagrafías óseas en individuos de etnia norteafricana y su relación con los niveles de APE y con la escala de Gleason obtenida en biopsia. *Actas Urológicas Españolas*, *35*(9), 534‑539. https://doi.org/10.1016/j.acuro.2011.03.013

Jazieh, A. R., Gaafar, R., Errihani, H., Jaafar, H., Al Dayel, F., Bahnassy, A. A., El Kadi, H., Abdallah, M. M., & Zaatari, G. (2021). Real-World Data on the Prevalence of Anaplastic Lymphoma Kinase–Positive Non–Small-Cell Lung Cancer in the Middle East and North Africa. *JCO Global Oncology*, (7), 1556‑1563. https://doi.org/10.1200/GO.21.00067

Jennane, S., Hasnaoui, N., Mahtat, E. M., Merimi, F., Bougar, S., El Maaroufi, H., Belmekki, A., Zafad, S., Essakalli, M., Mikdame, M., & Doghmi, K. (2020). Non-cryopreserved peripheral blood stem cells autologous transplantation in multiple myeloma : Bicentric study. *Transfusion Clinique et Biologique*, *27*(3), 152‑156. https://doi.org/10.1016/j.tracli.2020.03.006

Jouali, F., Marchoudi, N., Talbi, S., Bilal, B., El Khasmi, M., Rhaissi, H., & Fekkak, J. (2018). Detection of PIK3/AKT pathway in Moroccan population with triple negative breast cancer. *BMC Cancer*, *18*(1), 900. https://doi.org/10.1186/s12885-018-4811-x

Kaabouch, M., Chahdi, H., Azouzi, N., Oukabli, M., Rharrassi, I., Boudhas, A., Jaddi, H., Ababou, M., Dakka, N., Boichard, A., Bakri, Y., Dupuy, C., Al Bouzidi, A., & Ameziane El Hassani, R. (2020). BRAFV600E hot spot mutation in thyroid carcinomas : First Moroccan experience from a single-institution retrospective study. *African Health Sciences*, *20*(4), 1849‑1856. https://doi.org/10.4314/ahs.v20i4.40

Kaanane, H., El Attar, H., Louahabi, A., Berradi, H., Idrissi, H. H., Khyatti, M., & Nadifi, S. (2019). Targeted methods for molecular characterization of EGFR mutational profile in lung cancer Moroccan cohort. *Gene*, *705*, 36‑43. https://doi.org/10.1016/j.gene.2019.04.044

Kaanane, H., Senhaji, N., Berradi, H., Benchakroun, N., Benider, A., Karkouri, M., El Attar, H., Flores, O., Khyatti, M., & Nadifi, S. (2022). The influence of Interleukin-6, Interleukin-8, Interleukin-10, Interleukin-17, TNF-A, MIF, STAT3 on lung cancer risk in Moroccan population. *Cytokine*, *151*, 155806. https://doi.org/10.1016/j.cyto.2022.155806

Kaanane, H., Senhaji, N., Berradi, H., Benchakroun, N., Benider, A., Karkouri, M., El Attar, H., Igot Casa, Khyatti, M., & Nadifi, S. (2019). Association of Variants in IL6-Related Genes with Lung Cancer Risk in Moroccan Population. *Lung*, *197*(5), 601‑608. https://doi.org/10.1007/s00408-019-00261-0

Kabbaj, M., Oudghiri, M., Naya, A., Naamane, H., El Turk, J., Bennani, S., & Hassar, M. (2010). HLA-A, -B, -DRB1 alleles and haplotypes frequencies in Moroccan patients with leukemia. *Annales de Biologie Clinique*, *68*(3), 291‑296. https://doi.org/10.1684/abc.2010.0430

Kadouri, Y., Boualaoui, I., Lachkar, S., Sayegh, H., Benslimane, L., & Nouini, Y. (2020). Carcinome épidermoïde de la vessie : Expérience rétrospective dans un hôpital universitaire marocain et revue de la littérature. *Pan African Medical Journal*, *37*. https://doi.org/10.11604/pamj.2020.37.143.23540

Kaltoum, A. B. O., Sellama, N., Hind, D., Yaya, K., Mouna, L., & Asma, Q. (2020). MDR1 gene polymorphisms and acute myeloid leukemia AML susceptibility in A Moroccan adult population : A case-control study and meta-analysis. *Current Research in Translational Medicine*, *68*(1), 29‑35. https://doi.org/10.1016/j.retram.2019.06.001

Karkouri, M., Zafad, S., Khattab, M., Benjaafar, N., El Kacemi, H., Sefiani, S., Kettani, F., Dey, S., & Soliman, A. S. (2010). Epidemiologic profile of pediatric brain tumors in Morocco. *Child’s Nervous System*, *26*(8), 1021‑1027. https://doi.org/10.1007/s00381-010-1097-y

Kassogue, Y., Quachouh, M., Dehbi, H., Quessar, A., Benchekroun, S., & Nadifi, S. (2014). Functional polymorphism of CYP2B6 G15631T is associated with hematologic and cytogenetic response in chronic myeloid leukemia patients treated with imatinib. *Medical Oncology*, *31*(1), 782. https://doi.org/10.1007/s12032-013-0782-6

Khadiri, K., Khadrouf, Z., Mellouki, A., Bouchra, O., Essalihi, A., Naya, A., Benchekroun, M. T., & Karkouri, M. (2025). Epidemiological and molecular profile of breast cancer : A retrospective study in Casablanca, Morocco. *Pan African Medical Journal*, *50*. https://doi.org/10.11604/pamj.2025.50.105.43868

Khadrouf, Z., Khadiri, K., Mellouki, A., Essalihi, A., Bouchra, O., Benchekroun, M. T., Naya, A., & Karkouri, M. (2025). Epidemiological, pathological, and molecular characteristics of lung cancer in Casablanca, Morocco : A retrospective study. *Pan African Medical Journal*, *51*. https://doi.org/10.11604/pamj.2025.51.79.44198

Khalil, A. I., Bendahhou, K., Mestaghanmi, H., Saile, R., & Benider, A. (2016). Cancer du sein bilatéral synchrone : Expériences du centre Mohammed VI pour le traitement des cancers CHU Ibn Rochd Casablanca. *Pan African Medical Journal*, *25*. https://doi.org/10.11604/pamj.2016.25.121.9967

Khalil, J., Bellefqih, S., Afif, M., Elkacemi, H., Kebdani, T., & Benjaafar, N. (2015). Prognostic factors affecting cervical adenocarcinoma : 10 years experience in a single institution. *Archives of Gynecology and Obstetrics*, *292*(4), 915‑921. https://doi.org/10.1007/s00404-015-3701-6

Khalil, J., El Kacemi, H., Afif, M., Kebdani, T., & Benjaafar, N. (2015). Five years’ experience treating locally advanced cervical cancer with concurrent chemoradiotherapy : Results from a single institution. *Archives of Gynecology and Obstetrics*, *292*(5), 1091‑1099. https://doi.org/10.1007/s00404-015-3712-3

Khalis, M., Chajès, V., Moskal, A., Biessy, C., Huybrechts, I., Rinaldi, S., Dossus, L., Charaka, H., Mellas, N., Nejjari, C., Dorn, J., Soliman, A. S., Romieu, I., El Rhazi, K., & Charbotel, B. (2019). Healthy lifestyle and breast cancer risk : A case-control study in Morocco. *Cancer Epidemiology*, *58*, 160‑166. https://doi.org/10.1016/j.canep.2018.12.012

Khalis, M., Charbotel, B., Chajès, V., Rinaldi, S., Moskal, A., Biessy, C., Dossus, L., Huybrechts, I., Fort, E., Mellas, N., Elfakir, S., Charaka, H., Nejjari, C., Romieu, I., & El Rhazi, K. (2018). Menstrual and reproductive factors and risk of breast cancer : A case-control study in the Fez region, Morocco. *PLOS ONE*, *13*(1), e0191333. https://doi.org/10.1371/journal.pone.0191333

Khalis, M., Dossus, L., Rinaldi, S., Biessy, C., Moskal, A., Charaka, H., Fort, E., His, M., Mellas, N., Nejjari, C., Charbotel, B., Soliman, A. S., Romieu, I., Chajès, V., Gunter, M. J., Huybrechts, I., & El Rhazi, K. (2020). Body size, silhouette trajectory and the risk of breast cancer in a Moroccan case–control study. *Breast Cancer*, *27*(4), 748‑758. https://doi.org/10.1007/s12282-020-01072-5

Khalis, M., El Rhazi, K., Fort, E., Chajès, V., Charaka, H., Huybrechts, I., Moskal, A., Biessy, C., Romieu, I., Abbass, F., El Marnissi, B., Mellas, N., Nejjari, C., Soliman, A. S., & Charbotel, B. (2019). Occupation and risk of female breast cancer : A case‐control study in Morocco. *American Journal of Industrial Medicine*, *62*(10), 838‑846. https://doi.org/10.1002/ajim.23027

Khoubila, N., Bendari, M., Hda, N., Lamchahab, M., Qachouh, M., Rachid, M., & Quessar, A. (2019). Cytogenetic profile of a representative cohort of young adults with de novo acute myéloblastic leukaemia in Morocco. *Cancer Genetics*, *238*, 1‑9. https://doi.org/10.1016/j.cancergen.2019.06.010

Khtibari, Z., El Belhadji, M., Benhmidoune, L., Berrada, S., Rqibate, S., & Amraoui, A. (2015). Les carcinomes épidermoïdes des paupières. Bilan de 7ans d’expérience au service d’ophtalmologie adulte du CHU de Casablanca. *Journal Français d’Ophtalmologie*, *38*(2), 134‑140. https://doi.org/10.1016/j.jfo.2014.09.012

Kitab, B., Ezzikouri, S., Alaoui, R., Nadir, S., Badre, W., Trepo, C., Chemin, I., & Benjelloun, S. (2014). Occult HBV infection in Morocco : From chronic hepatitis to hepatocellular carcinoma. *Liver International*, *34*(6). https://doi.org/10.1111/liv.12482

Kriya, S., Omari, M., Nadi, S., El Asri, A., Mellas, N., Ragala, M. E. A., Amaadour, L., & Zarrouq, B. (2025a). Evaluation of religious coping strategies in women recently diagnosed with breast cancer in Morocco : Baseline findings from a cohort study. *BMJ Open*, *15*(6), e091991. https://doi.org/10.1136/bmjopen-2024-091991

Kriya, S., Omari, M., Nadi, S., El Asri, A., Mellas, N., Ragala, M. E. A., Amaadour, L., & Zarrouq, B. (2025b). Evaluation of religious coping strategies in women recently diagnosed with breast cancer in Morocco : Baseline findings from a cohort study. *BMJ Open*, *15*(6), e091991. https://doi.org/10.1136/bmjopen-2024-091991

Laabadi, K., Jayi, S., Alaoui, F. F., Bouguern, H., Chaara, H., Melhouf, M. A., Majdoub, K. I., Laalim, S. A., Anoun, H., Toughrai, I., & Mazaz, K. (2013). Cancer du sein de l’homme : À propos de 6 cas. *Pan African Medical Journal*, *16*. https://doi.org/10.11604/pamj.2013.16.70.2345

Laamiri, F. Z., Hasswane, N., Kerbach, A., Aguenaou, H., Taboz, Y., Benkirane, H., Mrabet, M., & Amina, B. (2016). Risk factors associated with a breast cancer in a population of Moroccan women whose age is less than 40 years : A case control study. *Pan African Medical Journal*, *24*. https://doi.org/10.11604/pamj.2016.24.19.8784

Laantri, N., Attaleb, M., Kandil, M., Naji, F., Mouttaki, T., Dardari, R., Belghmi, K., Benchakroun, N., El Mzibri, M., & Khyatti, M. (2011). Human papillomavirus detection in moroccan patients with nasopharyngeal carcinoma. *Infectious Agents and Cancer*, *6*(1), 3. https://doi.org/10.1186/1750-9378-6-3

Laantri, N., Jalbout, M., Khyatti, M., Ayoub, W. B., Dahmoul, S., Ayad, M., Bedadra, W., Abdoun, M., Mesli, S., Kandil, M., Hamdi‐Cherif, M., Boualga, K., Bouaouina, N., Chouchane, L., Benider, A., Ben‐Ayed, F., Goldgar, D., & Corbex, M. (2011). XRCC1 and hOGG1 genes and risk of nasopharyngeal carcinoma in North African countries. *Molecular Carcinogenesis*, *50*(9), 732‑737. https://doi.org/10.1002/mc.20754

Laaziri, K., Zyad, A., Laaziaf, E. M., Houdzi, J. E., Elhanafi, F., Brahim, I., Lakhouaja, N., Hazime, R., Ammara, M., Naya, A., & Admou, B. (2025). Acute Lymphoblastic Leukemia and Associated HLA-A, B, DRB1, and DQB1 Molecules : A Moroccan Pediatric Case–Control Study. *International Journal of Molecular Sciences*, *26*(11), 5295. https://doi.org/10.3390/ijms26115295

Lachgar, A., Toulba, A., & Kebdani, T. (2015). Service de radiothérapie de l’Institut National d’Oncologie- Rabat. Maroc. *LA TUNISIE MEDICALE*, *93*.

Laidi, F., Bouziane, A., Errachid, A., & Zaoui, F. (2016). Usefulness of Salivary and Serum Auto-antibodies Against Tumor Biomarkers HER2 and MUC1 in Breast Cancer Screening. *Asian Pacific Journal of Cancer Prevention*, *17*(1), 335‑339. https://doi.org/10.7314/APJCP.2016.17.1.335

Laidi, F., Bouziane, A., Lakhdar, A., Khabouze, S., Amrani, M., Rhrab, B., & Zaoui, F. (2014). Significant Correlation between Salivary and Serum Ca 15-3 in Healthy Women and Breast Cancer Patients. *Asian Pacific Journal of Cancer Prevention*, *15*(11), 4659‑4662. https://doi.org/10.7314/APJCP.2014.15.11.4659

Laidi, F., Bouziane, A., Lakhdar, A., Zaoui, F., Khabouze, S., & Rhrab, B. (2014). Salivary expression of soluble HER2 in breast cancer patients with positive and negative HER2 status. *OncoTargets and Therapy*, 1285. https://doi.org/10.2147/OTT.S64230

Lalya, I., Marnouche, E. A., Abdelhak, M., Zaghba, N., Andaloussi, K., Elmarjany, M., Baddouh, L., Dahmani, K., Hadadi, K., Sifat, H., & Mansouri, H. (2017). Radiotherapy of nasopharyngeal cancer using Rapidarc : Dosimetric study of military teaching hospital Mohamed V, Morocco. *BMC Research Notes*, *10*(1), 112. https://doi.org/10.1186/s13104-017-2430-2

Lamchahab, F. E., Tadlaoui, I., Beqqal, K., Bouattar, T., Ouzeddoun, N., Bayahia, R., Ait Ourhroui, M., Senouci, K., Hassam, B., & Ismaili, N. (2011). La maladie de Kaposi iatrogène au Maroc en dehors du contexte de la transplantation. *Annales de Dermatologie et de Vénéréologie*, *138*(11), 729‑735. https://doi.org/10.1016/j.annder.2011.06.007

Lamtai, H., Boutayeb, S., Mrabti, H., El Ghissassi, I., & Errihani, H. (2022). Cancer patients and COVID-19 vaccination, from safety to protocol adherence : A real-life setting report. *Frontiers in Oncology*, *12*, 1014786. https://doi.org/10.3389/fonc.2022.1014786

Lejbkowicz, F., Cohen, I., Barnett-Griness, O., Pinchev, M., Poynter, J., Gruber, S. B., & Rennert, G. (2012). Common MUTYH mutations and colorectal cancer risk in multiethnic populations. *Familial Cancer*, *11*(3), 329‑335. https://doi.org/10.1007/s10689-012-9516-8

Lemine Sow, M., El Yacoubi, H., Moukafih, B., Balde, S., Akimana, G., Najem, S., El Khoyaali, S., Abahssain, H., Chaibi, A., Zeb Khan, S., Trapani, D., Benzekri, A., Ghaouti, M., Gamra, L., Mestari, A., Kettani, F., Rahali, Y., Mrabti, H., Elghissassi, I., & Errihani, H. (2021). Frequency and types of *EGFR* mutations in Moroccan patients with non–small cell lung cancer. *Tumori Journal*, *107*(4), 335‑340. https://doi.org/10.1177/0300891620964571

Lkhoyaali, S., Haj, M. A. E., Khmamouche, M. R., Berrada, N., & Errihani, H. (2015). Service d’oncologie médicale – Institut National d’Oncologie Moulay Abdellah – Rabat – Maroc. *LA TUNISIE MEDICALE*, *93*.

Maadin, K., Majd, H., Demnati Sadki, N., Saoudi, M. T., El Meliani, O., Amaadour, L., Oualla, K., Benbrahim, Z., Mellas, N., & Arifi, S. (2025). Survival Outcomes and Prognostic Factors of Metastatic Breast Cancer in Elderly Women : A Retrospective Study in Northeastern Morocco. *Cureus*. https://doi.org/10.7759/cureus.91819

Madani, A., Zafad, S., Harif, M., Yaakoubi, M., Zamiati, S., Sahraoui, S., Benjelloun, A., Fehri, M., & Benchekroun, S. (2006). Treatment of Wilms tumor according to SIOP 9 protocol in Casablanca, Morocco. *Pediatric Blood & Cancer*, *46*(4), 472‑475. https://doi.org/10.1002/pbc.20436

Madihi, S., Laassili, C., Boukaira, S., Baha, W., Khyatti, M., Zyad, A., Ben Mkaddem, S., & Benani, A. (2024). Development and validation of the first HBV qRT-PCR assay in the Mediterranean area targeting the X region. *Journal of Virological Methods*, *326*, 114913. https://doi.org/10.1016/j.jviromet.2024.114913

Maghous, A., Rais, F., Ahid, S., Benhmidou, N., Bellahamou, K., Loughlimi, H., Marnouche, E., Elmajjaoui, S., Elkacemi, H., Kebdani, T., & Benjaafar, N. (2016). Factors influencing diagnosis delay of advanced breast cancer in Moroccan women. *BMC Cancer*, *16*(1), 356. https://doi.org/10.1186/s12885-016-2394-y

Majbar, M. A., Courtot, L., Dahbi-Skali, L., Rafik, A., Jouppe, P. O., Moussata, D., Benkabbou, A., Mohsine, R., Ouaissi, M., & Souadka, A. (2022). Two-step pull-through colo-anal anastomosis aiming to avoid stoma in rectal cancer surgery : A “real life” study in a developing country. *Journal of Visceral Surgery*, *159*(3), 187‑193. https://doi.org/10.1016/j.jviscsurg.2021.04.004

Mane, N., Lamchabbek, N., Mrah, S., Saidi, M., Elattabi, C., Faure, E., El M’rabet, F. Z., Najdi, A., Mellas, N., Bendahou, K., Belyamani, L., Saber, B., El Rhazi, K., Nejjari, C., Huybrechts, I., & Khalis, M. (2025). Anthropometry and the Risk of Breast Cancer in Moroccan Women : A Large Multicentric Case-Control Study. *Current Oncology*, *32*(8), 434. https://doi.org/10.3390/curroncol32080434

Marouf, C., Göhler, S., Filho, M. I. D. S., Hajji, O., Hemminki, K., Nadifi, S., & Försti, A. (2016). Analysis of functional germline variants in APOBEC3 and driver genes on breast cancer risk in Moroccan study population. *BMC Cancer*, *16*(1), 165. https://doi.org/10.1186/s12885-016-2210-8

Marouf, C., Hajji, O., Diakité, B., Tazzite, A., Jouhadi, H., Benider, A., & Nadifi, S. (2015). The CHEK2 1100delC allelic variant is not present in familial and sporadic breast cancer cases from Moroccan population. *SpringerPlus*, *4*(1), 38. https://doi.org/10.1186/s40064-014-0778-5

Marouf, C., Tazzite, A., Diakité, B., Jouhadi, H., Benider, A., & Nadifi, S. (2014). Association of TP53 PIN3 polymorphism with breast cancer in Moroccan population. *Tumor Biology*, *35*(12), 12403‑12408. https://doi.org/10.1007/s13277-014-2556-y

Maskrout, M., Boutaagount, F., Mokfi, R., Bennour, S., Senoussi, C., Mahlaq, S., Rais, F., & Rais, G. (2025). Exploring Human Epidermal Growth Factor Receptor 2 (HER2)-Low Early Breast Cancer in a Moroccan Population : Clinical Characteristics and Survival Outcomes. *Cureus*. https://doi.org/10.7759/cureus.90253

Mazti, A., El Idrissi, M., El Ibrahimi, A., Maaroufi, M. E., El Koubaiti, G., Bouhafa, T., El Fakir, S., Arifi, S., Mrini, A., & Chbani, L. (2021). How Can a Multidisciplinary Approach Improve Prognosis of Soft-Tissue Sarcomas of Extremities? *International Journal of Surgical Oncology*, *2021*, 1‑8. https://doi.org/10.1155/2021/8871557

McKay, J., Tenet, V., Franceschi, S., Chabrier, A., Gheit, T., Gaborieau, V., Chopin, S., Avogbe, P. H., Tommasino, M., Ainouze, M., Hasan, U., & Vaccarella, S. (2017). Immuno-related polymorphisms and cervical cancer risk : The IARC multicentric case-control study. *PLOS ONE*, *12*(5), e0177775. https://doi.org/10.1371/journal.pone.0177775

Mechita, N. B., Cherkaoui, S., Abousselham, L., Benmiloud, S., Kili, A., Kababri, M. E., Maani, K., Houdzi, J. E., Benajiba, N., Madani, A., Bennani, M., Belakhel, L., Bouffet, E., Patte, C., Harif, M., Youbi, M., & Hessissen, L. (2022). Implementing the WHO Global Initiative for Childhood Cancer in Morocco : Survival study for the six indexed childhood cancers. *Pediatric Blood & Cancer*, *69*(10), e29788. https://doi.org/10.1002/pbc.29788

Mellouki, I., Jellali, K., & Ibrahimi, A. (2018). Les tumeurs du gr�le : � propos de 27 cas. *Pan African Medical Journal*, *30*. https://doi.org/10.11604/pamj.2018.30.13.5407

Meryem, G., Sarah, N., Samia, G., Abdelkader, B., & Hassan, E. (s. d.). *Le traitement chirurgical des adénocarcinomes de la jonction œsogastrique : Expérience marocaine à travers une série de 149 cas*.

Mezouri, I., Chenna, H., Bellefqih, S., Elkacemi, H., Kebdani, T., & Benjaafar, N. (2014). Les perdus de vue en radiothérapie : Expérience de l’Institut National d’Oncologie au Maroc. *Pan African Medical Journal*, *19*. https://doi.org/10.11604/pamj.2014.19.18.4445

Mezzoug, N., Meski, F. Z., Khay, E. O., Skali Senhaji, N., Abrini, J., & Khattabi, A. (2025). Breaking Barriers in Cancer Screening : Understanding Participant Dropout in Breast and Cervical Programs in Morocco. *Asian Pacific Journal of Cancer Prevention*, *26*(1), 67‑75. https://doi.org/10.31557/APJCP.2025.26.1.67

Mimouni, M., Chaouki, W., Errihani, H., & Benjaafar, N. (2018). Analyse des délais de traitement du cancer du sein : Expérience d’un centre de référence tertiaire au Maroc. *Bulletin du Cancer*, *105*(9), 755‑762. https://doi.org/10.1016/j.bulcan.2018.05.010

Moufid, F. Z., Bouguenouch, L., El Bouchikhi, I., Chbani, L., Iraqui Houssaini, M., Sekal, M., Belhassan, K., Bennani, B., & Ouldim, K. (2018). The First Molecular Screening of *MLH1* and *MSH2* Genes in Moroccan Colorectal Cancer Patients Shows a Relatively High Mutational Prevalence. *Genetic Testing and Molecular Biomarkers*, *22*(8), 492‑497. https://doi.org/10.1089/gtmb.2018.0067

Moukafih, B., Abahssain, H., Mrabti, H., Errihani, H., Rahali, Y., Taoufik, J., & Chaibi, A. (2021). Impact of clinical pharmacy services in a hematology/oncology ward in Morocco. *Journal of Oncology Pharmacy Practice*, *27*(2), 305‑311. https://doi.org/10.1177/1078155220919169

Moumen, A., Dehbi, H., Kottwitz, D., El Amrani, M., Bouchoutrouch, N., El Hadi, H., Quessar, A., Benchekroun, S., Nadifi, S., & Sefrioui, H. (2015). Quantitative real-time polymerase chain reaction as an efficient molecular tool for detecting minimal residual disease in Moroccan chronic myeloid leukemia patients. *Genetics and Molecular Research*, *14*(1), 1044‑1055. https://doi.org/10.4238/2015.February.6.8

Mouna, B., Saber, B., Tijani, E. H., Hind, M., Amina, T., & Hassan, E. (2012). Primary malignant non-Hodgkin’s lymphoma of the breast : A study of seven cases and literature review. *World Journal of Surgical Oncology*, *10*(1), 151. https://doi.org/10.1186/1477-7819-10-151

Moundir, A., Ouair, H., Benhsaien, I., Jeddane, L., Rada, N., Amenzoui, N., Jouhadi, Z., Adnane, F., Hafidi, N. E., Kili, A., Bourhanbour Drissi, A., Babakhouya, A., Benmiloud, S., Hbibi, M., Benajiba, N., Hida, M., Bouskraoui, M., Mahraoui, C., Admou, B., … Bousfiha, A. A. (2023). Genetic Diagnosis of Inborn Errors of Immunity in an Emerging Country : A Retrospective Study of 216 Moroccan Patients. *Journal of Clinical Immunology*, *43*(2), 485‑494. https://doi.org/10.1007/s10875-022-01398-z

Moussaid, L., Benchikhi, H., Boukind, E. H., Sqalli, S., Mouaki, N., Kadiri, F., & Lakhdar, H. (2004). Tumeurs cutanées au cours du xeroderma pigmentosum au Maroc. *Annales de Dermatologie et de Vénéréologie*, *131*(1), 29‑33. https://doi.org/10.1016/S0151-9638(04)93538-7

Mrabti, H., Amziren, M., ElGhissassi, I., Bensouda, Y., Berrada, N., Abahssain, H., Boutayeb, S., El Fakir, S., Nejjari, C., Benider, A., Mellas, N., El Mesbahi, O., Bennani, M., Bekkali, R., Zidouh, A., & Errihani, H. (2016). Quality of life of early stage colorectal cancer patients in Morocco. *BMC Gastroenterology*, *16*(1), 131. https://doi.org/10.1186/s12876-016-0538-9

Mrabti, H., Sauvaget, C., Benider, A., Bendahhou, K., Selmouni, F., Muwonge, R., Alaoui, L., Lucas, E., Chami, Y., Villain, P., Abousselham, L., Carvalho, A. L., Bennani, M., Errihani, H., Sankaranarayanan, R., Bekkali, R., & Basu, P. (2021). Patterns of care of breast cancer patients in Morocco – A study of variations in patient profile, tumour characteristics and standard of care over a decade. *The Breast*, *59*, 193‑202. https://doi.org/10.1016/j.breast.2021.07.009

Nafil, H., Tazi, I., Sifsalam, M., Bouchtia, M., & Mahmal, L. (2012). Profil étiologique des pancytopénies chez l’adulte à Marrakech (Maroc). *Eastern Mediterranean Health Journal*, *18*(5), 532‑536. https://doi.org/10.26719/2012.18.5.532

Naim, A., Lahlou, Z., Kaanouch, O., Heddat, A., & Mansouri, S. (2024). Revolutionizing localized prostate cancer treatment : Stereotactic radiotherapy “Moroccan experience”. *Archivio Italiano Di Urologia e Andrologia*, *96*(1). https://doi.org/10.4081/aiua.2024.12104

Najdi, A., Berraho, M., Bendahhou, K., Obtel, M., Zidouh, A., Errihani, H., & Nejjari, C. (2014). Les déterminants du statut « perdu de vue » chez les patients pris en charge pour cancer au Maroc : Situation avant le Plan Cancer. *Pan African Medical Journal*, *18*. https://doi.org/10.11604/pamj.2014.18.83.2487

Omari, M., Kriya, S., Nadi, S., Zerhouni, G., Abbas, C. E., El Asri, A., Benbrahim, Z., Mellas, N., Rhazi, K. E., Amine Ragala, M. E., Hilaly, J. E., Halim, K., Amaadour, L., & Zarrouq, B. (2024). Effective and ineffective psychological adjustment in breast cancer patients before receiving neoadjuvant chemotherapy : Insights from a cohort study. *BMC Psychology*, *12*(1), 693. https://doi.org/10.1186/s40359-024-02211-9

Omari-Alaoui, H. E., Lahdiri, I., Nejjar, I., Hadadi, K., Ahyoud, F., Hachi, H., Alhilal, M., Errihani, H., Benjaafar, N., Souadka, A., & Gueddari, B. K. E. (s. d.). *Male breast cancer. A report of 71 cases Cancer du sein chez l’homme. À propos de 71 cas*.

Oumnia, B., Maladho, D., Amanda, G., Wafaa, K., Fadila, G., Mohamed, C., Najdi, A., Abderrahmane, A. B., Chakib, N., Nabil, I., & Mohamed, K. (2024). Immunohistochemical-Based Molecular Subtypes of Female Breast Cancer : A Retrospective Cross-Sectional Study at Cheikh Khalifa Hospital in Casablanca, Morocco. *Cancer Control*, *31*, 10732748241300655. https://doi.org/10.1177/10732748241300655

Ouzzif, Z., Doghmi, K., Messaoudi, N., Bouhsain, S., El Machtani, S., Biaz, A., Rachid, A., Dami, A., Bezza, A., & El Maataoui, A. (2023). Epidemiology of monoclonal gammopathy in Morocco – A hospital‐based study. *Cancer Reports*, *6*(5), e1814. https://doi.org/10.1002/cnr2.1814

Qarmiche, N., El Kinany, K., Otmani, N., El Rhazi, K., & Chaoui, N. E. H. (2023). Cluster analysis of dietary patterns associated with colorectal cancer derived from a Moroccan case–control study. *BMJ Health & Care Informatics*, *30*(1), e100710. https://doi.org/10.1136/bmjhci-2022-100710

Rabiou, S., Lakranbi, M., Ghizlane, T., Elfatemi, H., Serraj, M., Ouadnouni, Y., & Smahi, M. (2017). Quelle chirurgie pour quelle tumeur du médiastin : Expérience du service de chirurgie thoracique de CHU Hassan II de Fès. *Revue de Pneumologie Clinique*, *73*(5), 246‑252. https://doi.org/10.1016/j.pneumo.2017.04.001

Rahoui, J., Laraqui, A., Sbitti, Y., Touil, N., Ibrahimi, A., Ghrab, B., Al Bouzidi, A., Moussaoui Rahali, D., Dehayni, M., Ichou, M., Zaoui, F., & Mrani, S. (2014). Investigating the association of vascular endothelial growth factor polymorphisms with breast cancer : A Moroccan case–control study. *Medical Oncology*, *31*(9), 193. https://doi.org/10.1007/s12032-014-0193-3

Rahoui, J., Sbitti, Y., Touil, N., Laraqui, A., Ibrahimi, A., Rhrab, B., Al Bouzidi, A., Moussaoui Rahali, D., Dehayni, M., Ichou, M., Zaoui, F., & Mrani, S. (2014). The single nucleotide polymorphism +936 C/T VEGF is associated with human epidermal growth factor receptor 2 expression in Moroccan breast cancer women. *Medical Oncology*, *31*(12), 336. https://doi.org/10.1007/s12032-014-0336-6

Rais, G., Boutaagount, F., Mokfi, R., Maskrout, M., Bennour, S., Senoussi, C., Rais, F., & Lahlou, L. (2024). The Safety and Effectiveness of Bevacizumab in Metastatic Colorectal Cancer With Unresectable Metastases : A Real-Life Study From the South of Morocco. *Cureus*. https://doi.org/10.7759/cureus.56733

Rais, G., Raissouni, S., Aitelhaj, M., Rais, F., Naciri, S., Khoyaali, S., Abahssain, H., Bensouda, Y., Khannoussi, B., Mrabti, H., & Errihani, H. (2012). Triple negative breast cancer in Moroccan women : Clinicopathological and therapeutic study at the National Institute of Oncology. *BMC Women’s Health*, *12*(1), 35. https://doi.org/10.1186/1472-6874-12-35

Raissouni, S., Rais, G., Lkhoyaali, S., Aitelhaj, M., Mouzount, H., Mokrim, M., Razine, R., Kacemi, H. E., & Mrabti, H. (2013). *Clinical prognostic factors in locally advanced nasopharyngeal carcinoma in Moroccan population*. (14).

Ravery, V., Dominique, S., Hupertan, V., Ben Rhouma, S., Toublanc, M., Boccon-Gibod, L., & Boccon-Gibod, L. (2008). Prostate Cancer Characteristics in a Multiracial Community. *European Urology*, *53*(3), 533‑539. https://doi.org/10.1016/j.eururo.2007.04.048

Rebbani, K., Ezzikouri, S., Marchio, A., Kandil, M., Pineau, P., & Benjelloun, S. (2014). MDM2 285G>C and 344T>A gene variants and their association with hepatocellular carcinoma : A Moroccan case–control study. *Infectious Agents and Cancer*, *9*(1), 11. https://doi.org/10.1186/1750-9378-9-11

Reffai, A., Hori, M., Adusumilli, R., Bermudez, A., Haddad, H., Tawfiq, N., Pitteri, S., Bennani Mechita, M., & Mallick, P. (2025). Integrated Plasma and Tumor Proteomics of Nasopharyngeal Carcinoma in a Moroccan Cohort. *International Journal of Molecular Sciences*, *26*(12), 5771. https://doi.org/10.3390/ijms26125771

Rouibaa, F., Bakkar, M., Seddik, H., Addioui, T., Filali, F. Z., Akka, R., Desla, H., & Aourarh, A. (2013). Intérêt des prothèses métalliques expansives dans la prise en charge de l’occlusion tumorale colique : Expérience d’un service hospitalier Marocain. *Pan African Medical Journal*, *2*. https://doi.org/10.11604/pamj.2013.14.68.1982

S. Deoula, M., El Kinany, K., Huybrechts, I., Gunter, M. J., Hatime, Z., Boudouaya, H. A., Benslimane, A., Nejjari, C., El Abkari, M., Badre, W., El Feydi, A. E., Afkir, S., Abda, N., & El Rhazi, K. (2020). Consumption of meat, traditional and modern processed meat and colorectal cancer risk among the Moroccan population : A large‐scale case–control study. *International Journal of Cancer*, *146*(5), 1333‑1345. https://doi.org/10.1002/ijc.32689

Sakhi, Z., Najih, M., Elghazali, K., Tadlaoui, K. A., Benhessou, M., & Ennaji, M. M. (2025). Vitamin D receptor polymorphisms and their role in invasive breast cancer progression. *The Journal of Steroid Biochemistry and Molecular Biology*, *253*, 106819. https://doi.org/10.1016/j.jsbmb.2025.106819

Samlali, H., Assaid, N., Khobbaizi, Y., Hanicha, O., Abou El Houda, M., Nabil, S., Jouhadi, H., & Samlali, R. (2025). High-Dose-Rate Brachytherapy Boost for Prostate Cancer : A Retrospective Observational Study in Low- and Middle-Income Countries. *Advances in Radiation Oncology*, *10*(10), 101861. https://doi.org/10.1016/j.adro.2025.101861

Sasco, A. J., Merrill, R. M., Dari, I., & Bartal, M. (s. d.). *A case–control study of lung cancer in Casablanca, Morocco*.

Sbitti, Y., Kadiri, H., Essaidi, I., Fadoukhair, Z., Kharmoun, S., Slimani, K., Ismaili, N., Ichou, M., & Errihani, H. (2011). Breast cancer treatment and sexual dysfunction : Moroccan women’s perception. *BMC Women’s Health*, *11*(1), 29. https://doi.org/10.1186/1472-6874-11-29

Schairer, C., Hablas, A., Eldein, I. A. S., Gaafar, R., Rais, H., Mezlini, A., Ayed, F. B., Ayoub, W. B., Benider, A., Tahri, A., Khouchani, M., Aboulazm, D., Karkouri, M., Eissa, S., Bastawisy, A. E., Yehia, M., Gadalla, S. M., Swain, S. M., Merajver, S. D., … Soliman, A. S. (2020). Risk factors for inflammatory and non-inflammatory breast cancer in North Africa. *Breast Cancer Research and Treatment*, *184*(2), 543‑558. https://doi.org/10.1007/s10549-020-05864-3

Schairer, C., Hablas, A., Eldein, I. A. S., Gaafar, R., Rais, H., Mezlini, A., Ayed, F. B., Ayoub, W. B., Benider, A., Tahri, A., Khouchani, M., Aboulazm, D., Karkouri, M., Eissa, S., Pfeiffer, R. M., Gadalla, S. M., Swain, S. M., Merajver, S. D., Brown, L. M., & Soliman, A. S. (2019). Clinico-pathologic and mammographic characteristics of inflammatory and non-inflammatory breast cancer at six centers in North Africa. *Breast Cancer Research and Treatment*, *176*(2), 407‑417. https://doi.org/10.1007/s10549-019-05237-5

Sekal, M., Ameurtesse, H., Chbani, L., Ouldim, K., Bennis, S., Abkari, M., Boulouz, A., Benajah, D. A., Benjelloun, B., Ousadden, A., Ait Taleb, K., Ait Laalim, S., Toghrai, I., Mazaz, K., Arifi, S., Mellas, N., El Rhazi, K., Harmouch, T., Ibrahimi, S. A., & Amarti Riffi, A. (2015). Epigenetics could explain some Moroccan population colorectal cancers peculiarities : Microsatellite instability pathway exploration. *Diagnostic Pathology*, *10*(1), 77. https://doi.org/10.1186/s13000-015-0326-9

Sekkat, H., Souadka, A., Courtot, L., Rafik, A., Amrani, L., Benkabbou, A., Peyrafort, P., Giger-Pabst, U., Karam, E., Mohsine, R., Majbar, A. M., & Ouaissi, M. (2022). Available prediction scores of conversion for laparoscopic rectal cancer surgery seem to be unsuitable for nowadays rectal cancer management. *BMC Surgery*, *22*(1), 162. https://doi.org/10.1186/s12893-022-01617-9

Sekkate, S., Kairouani, M., Serji, B., Tazi, A., Mrabti, H., Boutayeb, S., & Errihani, H. (s. d.). *Ovarian granulosa cell tumors : A retrospective study of 27 cases and a review of the literature*.

Selmouni, F., Bendahhou, K., Sauvaget, C., Abahssain, H., Lucas, E., Muwonge, R., Mimouni, H., Ismaili, R., Bidar, S., Benkaddour, F. Z., Abousselham, L., Chami Khazraji, Y., Belakhel, L., & Basu, P. (2024). Impact of clinical breast examination‐based screening program on care pathway, stage at diagnosis, nature of treatment, and overall survival among breast cancer patients in Morocco. *Cancer*, *130*(19), 3353‑3363. https://doi.org/10.1002/cncr.35419

Senhaji, L., Abbassi, M., Senhaji, N., Benmaamar, S., Dani, L., El Rhazi, K., Karhate, M., Serraj, M., El Biaze, M., Benjelloun, M. C., Ouldim, K., Bouguenouch, L., & Amara, B. (2025). MUC5B Polymorphism and Susceptibility to Idiopathic Pulmonary Fibrosis in Morocco. *Cureus*. https://doi.org/10.7759/cureus.86806

Senhaji, N., Louati, S., Chbani, L., Bardai, S. E., Mikou, K., Maaroufi, M., Benzagmout, M., Faiz, M. C. E., Marie, Y., Mokhtari, K., Idbaih, A., & Amarti, A. (2016). Prevalence of IDH1/2 Mutations in Different Subtypes of Glioma in the North-East Population of Morocco. *Asian Pacific Journal of Cancer Prevention*, *17*.

Skhoun, H., Khattab, M., Belkhayat, A., Takki Chebihi, Z., Bakri, Y., Dakka, N., & El Baghdadi, J. (2022). Association of TP53 gene polymorphisms with the risk of acute lymphoblastic leukemia in Moroccan children. *Molecular Biology Reports*, *49*(9), 8291‑8300. https://doi.org/10.1007/s11033-022-07643-3

Slaoui, A., Jabbour, Y., Ghazoui, A. E., Karmouni, T., Elkhader, K., Koutani, A., & Attaya, A. I. (2015). Penile cancer : About ten cases at the University Hospital of Rabat, review of the literature. *Pan African Medical Journal*, *22*. https://doi.org/10.11604/pamj.2015.22.53.6563

Slaoui, M., Mouh, F. Z., Ghanname, I., Razine, R., El Mzibri, M., & Amrani, M. (2016). Outcome of Breast Cancer in Moroccan Young Women Correlated to Clinic-Pathological Features, Risk Factors and Treatment : A Comparative Study of 716 Cases in a Single Institution. *PLOS ONE*, *11*(10), e0164841. https://doi.org/10.1371/journal.pone.0164841

Slaoui, M., Zoure, A. A., Mouh, F. Z., Bensouda, Y., El Mzibri, M., Bakri, Y., & Amrani, M. (2018). Outcome of inflammatory breast cancer in Moroccan patients : Clinical, molecular and pathological characteristics of 219 cases from the National Oncology Institute (INO). *BMC Cancer*, *18*(1), 713. https://doi.org/10.1186/s12885-018-4634-9

Smith, J. S., Bosetti, C., Muñoz, N., Herrero, R., Bosch, F. X., Eluf‐Neto, J., Meijer, C. J. L. M., Van Den Brule, A. J. C., Franceschi, S., & Peeling, R. W. (2004). *Chlamydia trachomatis* and invasive cervical cancer : A pooled analysis of the IARC multicentric case‐control study. *International Journal of Cancer*, *111*(3), 431‑439. https://doi.org/10.1002/ijc.20257

Soliman, A. S., & Schairer, C. (2012). Considerations in setting up and conducting epidemiologic studies of cancer in middle‐ and low‐income countries : The experience of a case–control study of inflammatory breast cancer in N orth A frica in the past 10 years. *Cancer Medicine*, *1*(3), 338‑349. https://doi.org/10.1002/cam4.36

Soro, S., Amalou, G., Benchikh, S., El Hamouchi, A., Charoute, H., Oukheda, M., Abdelouaheb, B. B., Saile, R., Lebrazi, H., & Nassereddine, S. (2024). Molecular Profile of BCR-ABL1 Negative Myeloproliferative Neoplasm in a Moroccan Population. *Asian Pacific Journal of Cancer Prevention*, *25*(11), 4013‑4025. https://doi.org/10.31557/APJCP.2024.25.11.4013

Souadka, A., Habbat, H., Makni, A., Abid, M., El Mouatassim, Z., Daghfous, A., Korjani, Z., Rebai, W., Ayadi, M., Messai, W. H., Majbar, M. A., Benkabbou, A., Mohsine, R., & Souadka, A. (2025). Advancing Treatment Outcomes for Peritoneal Surface Malignancies in Low- and Middle-Income Countries : Insights from the First Multicenter Study in North Africa. *Cancers*, *17*(13), 2113. https://doi.org/10.3390/cancers17132113

Soughi, M., Meziane, M., Gallouj, S., & Mernissi, F. (2019). Étude descriptive dermoscopique d’une série de 100 carcinome basocellulaires diagnostiqués au Maroc. *Pan African Medical Journal*, *34*. https://doi.org/10.11604/pamj.2019.34.64.6377

Soumia, F., Leila, A., Mohamed, R., Laila, H., & Mustapha, E. (2017). Intérêt de la TEP-TDM dans le cancer broncho-pulmonaire primitif non à petite cellule. *Pan African Medical Journal*, *28*. https://doi.org/10.11604/pamj.2017.28.289.13130

Tabiti, H., Gbadamassi, A. A., Bendahhou, K., Oussafrar, Z., & Guensi, A. (2025). Occurrence of Metastases in Differentiated Thyroid Carcinoma Patients : A Retrospective Study in Morrocco Covering 10 Years of Follow-Up. *Cureus*. https://doi.org/10.7759/cureus.78176

Tabiti, H., Guensi, A., & Bendahhou, K. (2025). Long-Term Survival Rate for Moroccan Patients with Differentiated Thyroid Cancer. *Asian Pacific Journal of Cancer Prevention*, *26*(3), 913‑923. https://doi.org/10.31557/APJCP.2025.26.3.913

Tabyaoui, I., Maani, K. E., & Othmani, M. B. (2013). *High Incidence of MYCN Amplification in a Moroccan Series of Neuroblastic Tumors : Comparison to Current Biological Data*. *22*(2).

Tabyaoui, I., Tahiri-Jouti, N., Serhier, Z., Bennani-Othmani, M., Sibai, H., Itri, M., Benchekroun, S., & Zamiati, S. (2013). Immunohistochemical expression of CD44s in human neuroblastic tumors : Moroccan experience and highlights on current data. *Diagnostic Pathology*, *8*(1), 39. https://doi.org/10.1186/1746-1596-8-39

Tafenzi, H. A., Choulli, F., Essaadi, I., & Belbaraka, R. (2025). Real-World Outcomes of Combination Anthracycline and Taxane Adjuvant Therapies in Early Triple-Negative Breast Cancer : A Moroccan Retrospective Analysis. *JCO Global Oncology*, (11), e2400650. https://doi.org/10.1200/GO-24-00650

Tafenzi, H. A., Choulli, F., Essadi, I., & Belbaraka, R. (2025). Second-line chemotherapy rechallenge in lung cancer patients : A Moroccan real-world study. *Frontiers in Oncology*, *15*, 1489327. https://doi.org/10.3389/fonc.2025.1489327

Tafenzi, H. A., Choulli, F., Haag, E. K., Baladi, A., Essaadi, I., & Belbaraka, R. (2024). Real world results of locally advanced and metastatic lung cancer patients treated with platinum doublet chemotherapy in first line : Moroccan cohort. *Translational Oncology*, *47*, 102015. https://doi.org/10.1016/j.tranon.2024.102015

Tanouti, I.-A., Fellah, H., El Fihry, R., Zerrad, C., Abounouh, K., Tahiri, M., Belkouchi, A., Badre, W., Pineau, P., Benjelloun, S., & Ezzikouri, S. (2023). Association of Peroxisome Proliferator-Activated Receptor Gamma Coactivator 1 Alpha Coding Variants with Hepatocellular Carcinoma Risk in the Moroccan Population : A Case-Control Study. *Asian Pacific Journal of Cancer Prevention*, *24*(11), 3689‑3696. https://doi.org/10.31557/APJCP.2023.24.11.3689

Tanz, R., Mahfoud, T., Bazine, A., Aassab, R., Benjaafar, N., Gueddari, B. E. K. E., Ichou, M., & Errihani, H. (2012). Endometrial stromal sarcoma : Prognostic factors and impact of adjuvant therapy in early stages. *Hematology/Oncology and Stem Cell Therapy*, *5*(1), 31‑35. https://doi.org/10.5144/1658-3876.2012.31

Tanz, R., Mahfoud, T., Bazine, A., Khmamouch, R., Bensouda, Y., Ismaili, N., Benjaafar, N., El Gueddari, B. K., Ichou, M., & Errihani, H. (2011). Tolérance cardiaque du trastuzumab en adjuvant : Revue à travers 53 observations. *Journal de Gynécologie Obstétrique et Biologie de la Reproduction*, *40*(2), 144‑148. https://doi.org/10.1016/j.jgyn.2010.12.003

Taoufiq, N., Naim, A., Bouchbika, Z., Benchekroune, N., Jouhadi, H., Sahraoui, S., & Benider, A. (2017). Tumeurs Stromales Gastro-Intestinales «GIST» : État des lieux et actualités à travers notre expérience portant sur 54 cas et une Revue de littérature. *Pan African Medical Journal*, *27*. https://doi.org/10.11604/pamj.2017.27.165.7754

Tarwate, M., Benchikhi, H., Adarmouch, L., Benider, A., Amine, M., Zamiati, S., & Boukind, E. H. (2014). Mélanome cutané épais : Facteurs de mortalité et de survenue de métastases. *Pan African Medical Journal*, *18*. https://doi.org/10.11604/pamj.2014.18.44.2812

Tazi, I., Nafil, H., Mahmal, L., Harif, M., Khouchani, M., Saadi, Z., Belbaraka, R., Elomrani, A., & Tahri, A. (2013). Les médecines alternatives et complémentaires chez les patients cancéreux en cours de traitement à Marrakech, Maroc : Étude prospective. *Bulletin de la Société de pathologie exotique*, *106*(4), 278‑285. https://doi.org/10.1007/s13149-013-0308-7

Tazzite, A., Jouhadi, H., Saiss, K., Benider, A., & Nadifi, S. (s. d.). *RELATIONSHIP BETWEEN FAMILY HISTORY OF BREAST CANCER AND CLINICOPATHOLOGICAL FEATURES IN MOROCCAN PATIENTS*.

Tazzite, A., Kassogue, Y., Diakité, B., Jouhadi, H., Dehbi, H., Benider, A., & Nadifi, S. (2016). Association between ABCB1 C3435T polymorphism and breast cancer risk : A Moroccan case-control study and meta-analysis. *BMC Genetics*, *17*(1), 126. https://doi.org/10.1186/s12863-016-0434-x

Tissir, R., Lamchahab, M., Benhassou, M., Quachouh, M., Rachid, M., & Benchakroun, S. (s. d.). *Difficulté de la prise en charge de la Leucémie aiguë au cours de la grossesse au Maroc*.

Titou, H., Kerrouch, H., Frikh, R., & Hjira, N. (2022). The association between bullous pemphigoid and comorbidities : A case-control study in Moroccan patients. *Acta Dermatovenerologica Alpina Pannonica et Adriatica*, *31*(1). https://doi.org/10.15570/actaapa.2022.2

Touati, M. M., Aljalil, A., Darouassi, Y., Chihani, M., Lahkim, M., Fassi, J. A., Bouaity, B., & Ammar, H. (2015). Les carcinomes de la thyroïde : Profils épidémiologique, clinique et thérapeutique, à propos de 102 cas. *Pan African Medical Journal*, *21*. https://doi.org/10.11604/pamj.2015.21.59.5688

Traore, B. M., El Fakir, S., Charaka, H., Benaicha, N., Najdi, A., Zidouh, A., Bennani, M., Errihani, H., Mellass, N., Benider, A., Bekkali, R., & Nejjari, C. (2018). Evolution of quality of life in patients with breast cancer during the first year of follow-up in Morocco. *BMC Cancer*, *18*(1), 109. https://doi.org/10.1186/s12885-018-4008-3

Zerrad, C., Lkhider, M., Bouqdayr, M., Belkouchi, A., Badre, W., Tahiri, M., Pineau, P., Benjelloun, S., & Ezzikouri, S. (2024). NOD1 and NOD2 genetic variants : Impact on hepatocellular carcinoma susceptibility and progression in Moroccan population. *Gene*, *931*, 148847. https://doi.org/10.1016/j.gene.2024.148847

Zineb Aoullay, Meriem Slaoui, Rachid Razine, Abdelouahed Er-Raki, Bouchra Meddah, & Yahia Cherrah. (2020). Therapeutic Characteristics, Chemotherapy-Related Toxicities and Survivorship in Colorectal Cancer Patients. *Ethiopian Journal of Health Sciences*, *30*(1). https://doi.org/10.4314/ejhs.v30i1.9

Znati, K., Bennis, S., Abbass, F., Akasbi, Y., Chbani, L., Elfatemi, H., Harmouch, T., & Amarti, A. (2014). Cancer du sein chez la femme jeune dans le Nord-Est du Maroc. *Gynécologie Obstétrique & Fertilité*, *42*(3), 149‑154. https://doi.org/10.1016/j.gyobfe.2011.08.014

Znati, K., Chahbouni, S., Hammas, N., Bennis, S., Abbas, F., Harmouch, T., Chbani, L., Elfatemi, H., & Amarti, A. (2011). Twelve cases of metaplastic carcinoma of the breast : Experience of the university hospital of Fez Morocco. *Archives of Gynecology and Obstetrics*, *283*(4), 845‑849. https://doi.org/10.1007/s00404-010-1474-5

Zouine, S., Marnissi, F., Otmani, N., Bennani Othmani, M., El Wafi, M., Kojok, K., Zaid, Y., Tahiri Jouti, N., & Habti, N. (2016). ABO blood groups in relation to breast carcinoma incidence and associated prognostic factors in Moroccan women. *Medical Oncology*, *33*(7), 67. https://doi.org/10.1007/s12032-016-0784-2
